# Construction and validation of an immune-related genes prognostic index (IRGPI) model in colon cancer

**DOI:** 10.3389/fendo.2022.963382

**Published:** 2022-11-09

**Authors:** Yabin Jin, Jianzhong Deng, Bing Luo, Yubo Zhong, Si Yu

**Affiliations:** ^1^ Institute of Clinical Research, The First People’s Hospital of Foshan, Foshan, China; ^2^ Department of Anorectal Surgery, The First People’s Hospital of Foshan, Foshan, China; ^3^ Department of Gastrointestinal Surgery, The Second People’s Hospital of Foshan, Foshan, China; ^4^ Division of Gastrointestinal Surgery, Department of Surgery, The University of Hong Kong-Shenzhen Hospital, Shenzhen, China

**Keywords:** colon cancer, immune cells, prognostic model, biomarker, tumor microenvironment (TME)

## Abstract

**Background:**

Though immunotherapy has become one of the standard therapies for colon cancer, the overall effective rate of immunotherapy is very low. Constructing an immune-related genes prognostic index (IRGPI) model may help to predict the response to immunotherapy and clinical outcomes.

**Methods:**

Differentially expressed immune-related genes (DEIRGs) between normal tissues and colon cancer tissues were identified and used to construct the co-expression network. Genes in the module with the most significant differences were further analyzed. Independent prognostic immune-related genes (IRGs) were identified by univariate and multivariate cox regression analysis. Independent prognostic IRGs were used to construct the IRGPI model using the multivariate cox proportional hazards regression model, and the IRGPI model was validated by independent dataset. ROC curves were plotted and AUCs were calculated to estimate the predictive power of the IRGPI model to prognosis. Gene set enrichment analysis (GSEA) was performed to screen the enriched KEGG pathways in the high-risk and low-risk phenotype. Correlations between IRGPI and clinical characteristic, immune checkpoint expression, TMB, immune cell infiltration, immune function, immune dysfunction, immune exclusion, immune subtype were analyzed.

**Results:**

Totally 680 DEIRGs were identified. Three independent IRGs,NR5A2, PPARGC1A and LGALS4, were independently related to survival. NR5A2, PPARGC1A and LGALS4 were used to establish the IRGPI model. Survival analysis showed that patients with high-risk showed worse survival than patients in the low-risk group. The AUC of the IRGPI model for 1-year, 3-year and 5-year were 0.584, 0.608 and 0.697, respectively. Univariate analysis and multivariate cox regression analysis indicated that IRGPI were independent prognostic factors for survival. Stratified survival analysis showed that patients with IRGPI low-risk and low TMB had the best survival, which suggested that combination of TMB and IRGPI can better predict clinical outcome. Immune cell infiltration, immune function, immune checkpoint expression and immune exclusion were different between IRGPI high-risk and low-risk patients.

**Conclusion:**

An immune-related genes prognostic index (IRGPI) was constructed and validated in the current study and the IRGPI maybe a potential biomarker for evaluating response to immunotherapy and clinical outcome for colon cancer patients.

## Introduction

Colon cancer is one of the most common gastrointestinal malignancies worldwide, with the fourth highest incidence among all types of cancers, and is the second leading cause of cancer-related death (1). Due to the screening programs, the incidence and mortality of colon cancer has been decreased. Advancements in treatments, including chemotherapy, target therapy and immunotherapy, the prognosis of colon cancer was improved (2). However, the overall survival of colon cancer is still poor, with a 5-year survival rate of approximately 64% (2). Thus, it is an essential need to identify new diagnostic, prognostic and therapeutic biomarker for colon cancer.

Dysregulation of immune system is involved in the occurrence and development of tumor and immunotherapy has become one of the standard therapies for solid tumors, including colon cancer (3, 4). Immune checkpoint inhibitors, such as anti-PD-1/PD-L1 antibodies, are the most widely used immunotherapy in colon cancer treatment. However, the overall effective rate of anti-PD-1/PD-L1 antibodies is low, with an overall response rate (ORR) of 30%-50% in colorectal cancer patients with high microsatellite instability (MSI-H) and 0% in patients with microsatellite stability (MSS) (5, 6). The reason of the low efficacy maybe due to low tumor immunogenicity, low infiltration level of CD8+T cells and low positive rate of PD-1 (7–10). Identification of the immune-related genes (IRGs) in colon cancer may help to predict the response to immune checkpoint therapy and optimize treatment.

In recent years, new biomarkers have been identified in a variety of tumors through the analysis of public database, including cyclin D1 (11), RBP7 (12), Heat shock protein beta 3 (HSPB3) (13), etc. Several prognostic models have also been constructed for some types of cancers. For instance, an immune cell infiltration (ICI) score model was identified and validated for colon cancer base on TCGA database (14). Several IRGs prognostic models have been reported for thyroid cancer (15), gastric cancer (16) and ovarian cancer (17). A prognostic index based on 7 IRGs was constructed in papillary thyroid cancer (PTC) and the prognostic index was shown to be an independent predictor for clinical outcome. High-risk of IRGs prognostic index was related to poor survival (15). Yang et al. constructed a predictive model in gastric cancer patients based on 10 IRGs. The predictive model showed a powerful efficiency in distinguishing good or poor survival of gastric cancer patients (16). A 7 IRGs signatures established by Geng et al. demonstrated high reliability and feasibility in predicting the prognosis of Helicobacter pylori-Infected gastric cancer, and also helped to predict sensitivity to anti-PD-1 therapy (18).

In the current study, we aimed to establish an immune-related gene prognostic index (IRGPI) for colon cancer. Firstly, we screened the IRGs with differential expression between normal tissues and colon cancer tissues base on the TCGA-COAD (https://portal.gdc.cancer.gov/) dataset. Totally 680 IRGs were identified. Then a weighted gene co-expression network analysis (WGCNA) was performed to identify the core modules and central IRGs. Among the module genes with the most significant difference between normal tissues and tumor tissues, seven IRGs were related to survival. Three IRGs, NR5A2, PPARGC1A, LGALS4, were included to constructe the prognostic index using a multivariate cox proportional hazards regression analysis. The predictive power of the prognostic index was validated by an independent dataset from GEO database. The correlation of the IRGPI with clinical characteristics was also analyzed. The IRGPI constructed in the current study is a potential biomarker for prognosis and may help to predict response to immune checkpoint therapy in colon cancer patients.

## Materials and methods

### Data resource

Gene expression data and clinical information of colon cancer patients (TCGA-COAD) were downloaded from The Cancer Genome Atlas (https://portal.gdc.cancer.gov/) database (up to September, 2021, filter conditions were showed in the workflow chart in **Supplementary Material S1**) and used as training set. Datasets with gene expression information, survival information and free annotation files (GSE17536, GSE17537, GSE29621, GSE40967) from Gene Expression Omnibus (https://www.ncbi.nlm.nih.gov/geo/) database were used as validation set. Data were analyzed by R x64 software (v4.0.5) and Strawberry-perl software (v5.32.0). The batch effects between different datasets were adjusted by the ComBat algorithm (19). Totally 2660 genes were identified as IRGs using the InnateDB (20) (http://www.innatedb.com) and Immport database (21) (https://www.immport.org/home).

### R and perl packages/libraries used in the study

The ComBat algorithm (19) was used to adjust the batch effects between different datasets. The Limma R package (22) was used to analysis the gene expression difference between different groups. The ClusterProfiler R package (23) was used to analyze the enriched KEGG pathway ways and GO pathways. WGCNA R package (24) was used to construct the co-expression network. The Maftool R package (25) was used to calculate the mutation burden. The CIBERSORT algorithm (26) was used to analyze the immune cell infiltrations in tumor microenvironment.

### Analysis of IRGs expression difference

Immune-related genes expression difference between normal tissues and colon cancer tissues were analyzed using limma package (22). Difference was considered as significant if adjusted p<0.05 and absolute value of fold-change >1, which means that the gene expression difference is more than 1 times (26). ClusterProfiler R package (23) was used to analyze the functionally enriched genes in different Kyoto encyclopedia of genes and genomes (KEGG) pathways and gene ontology (GO) pathways. Results were considered as significant if false discovery rate (FDR)<0.05. Results were visualized by chord diagrams.

### Construction of co-expression network

WGCNA R package (24) was used to construct the co-expression network. An adjacency matrix was established to analysis the correlation strength between different genes. The soft-threshold (β) calculated by the WGCNAR was 6. Then, the adjacency matrix was transformed into a topological overlap matrix (TOM). Next, hierarchical clustering was performed to identify modules. Each module contains at least 25 genes. Finally, modules eigengene was calculated, modules were hierarchically clustered and similar modules were merged (abline=0.25). Module with the smallest p values was considered as module with the most significant difference between normal and tumor tissues. Genes included in this module were further analyzed.

### Construction and verification of immune-related genes prognostic index model

Genes in the module with the most significant difference between normal and tumor tissues were further analyzed. Seven IRGs were identified as prognostic genes by univariate cox regression analysis. Then multivariate cox proportional hazards regression was performed to construct a prognostic index model. The prognostic index was calculated as follow:


Prognostic index=∑regression coefficient(IRGi)×expression (IRGi)

Patients in the training set were classified into high-risk and low-risk groups based on the median value of the prognostic index. Survival of the high-risk and low-risk groups was compared by Kaplan-Meier method. The ROC was plotted and area under curve (AUC) was calculated to assess the predictive power of the prognostic index. Univariate and multivariate cox regression analysis were performed to estimate the independence of the prognostic index to survival. Patients in GEO datasets were used as a validating set to verify the prognostic index model.

### Gene set enrichment analysis

Gene set enrichment analysis (GSEA) was performed to screen the enriched KEGG pathways in the high-risk and low-risk phenotypes using the c2.cp.kegg.v6.2.symbols.gmt (27)as reference gene set. Results were considered as significant if FDR<0.05.

### Analysis of clinical and immune characteristics

Nucleotide variation leads to gene mutation. Based on the nucleotide variation information extracted from TCGA database, the numbers of mutation of high-risk and low-risk group were calculated using the Maftool package (25). The immune cell infiltrations in high-risk and low-risk were estimated by CIBERSORT algorithm (28). The scores of different immune functions were calculated based on the immune-related biomarkers, including infiltration of different immune cells(dendritic cells, B cells, macrophages, mast cells, neutrophils, NK cells, Th1 cells, Th2 cells, Tfh cell, Treg cells), APC co-inhibition, APC cos-stimulation, CCR, immune checkpoint, cytolytic activity, HLA, inflammation promoting, MHC I, MHC II, parainflammation, T cell co-inhibition, T cell co-stimulation, Type I and Type II IFN response (29). The T cell dysfunction was charactered by high infiltration level of cytotoxic T lymphocytes (CTL) and the prevention of T cell infiltration in tumors. Immune dysfunction and exclusion scores of tumors can be evaluated by the expression of gene signatures constructed by Peng J et al (30). Immune dysfunction and exclusion scores of different cancer cohorts in TCGA database have been calculated and uploaded to the TIDE database (http://tide.dfci.harvard.edu/). We downloaded the immune dysfunction and exclusion score of TCGA-COAD from TIDE database. Patients were classified into three subtype, C1 (wound healing), C2 (IFN-γ dominant) and C4 (lymphocyte depleted) based on non-negative matrix factorization (NMF) clustering analysis (31). Correlations between IRG prognostic index and clinical characteristic, immune checkpoint expression, TMB, immune cell infiltration, immune function, immune dysfunction, immune exclusion, immune subtype were analyzed.

### qRT-PCR analysis of gene expression in colon cancer cell lines

qRT-PCR was used to examine the IRGs expression level *in vitro*. Colon cancer cell lines (HCT116 and COLO320) and normal colonic epithelial cell line(CP-H040) were purchased from ATCC cell bank. Cells were cultured in RPMI-1640 median (Procell, Wuhan, China). Total RNA was extracted using Trizol reagent (Invitrogen, USA). RT-PCR kit (Takara, Japan) was used to perform reverse transcription, according to the manufacturer’s instruction. qRT-PCR was performed using the SYBR Green reagent (ABI, USA). Primer sequences used in the qRT-PCR were shown as follows: (1) NR5A2 forward: 5’-AAGAAAGCCCTCATTCGAG-3’; NR5A2 reverse: 5’-TGTTCCGGTTATTCTGCTC-3’; (2) PPARGC1A forward: 5’-CCTGCATGAGTGTGTGCTCT-3; PPARGC1A reverse: 5’-CTCAGAGTCCTGGTTGCACA-3’; (3) LGALS4 forward: 5’-CGAGGAGAAGAAGATCACCC-3’; LGALS4 reverse: 5’-CTCTGGAAGGCCGAGAGG-3’; (4) β−actin forward: 5’-GATGAGATTGGCATGGCTTT-3’, β−actin reverse: 5’-CACCTTCACCGTTCCAGTTT-3’.

### Statistical analysis

Statistical analyses were performed using R software (R x64 version 4.0.5). Comparison of two independent samples was tested by Wilcoxon test. Comparison of multiple independent samples was tested by Kruskal-Wallis test. Survival difference of Kaplan-Meier curves were compared by log-rank test. Correlation between prognostic index and other factors was analyzed by Spearman correlation analysis. Cox regression analysis was performed to identify the prognostic factors correlated with survival. Comparison of clinicopathological features in high-risk and low-risk group was estimated by Chi square test. Results were considered as significant if p<0.05.

## Results

### Identification of differentially expressed IRGs

**Figure 1:**
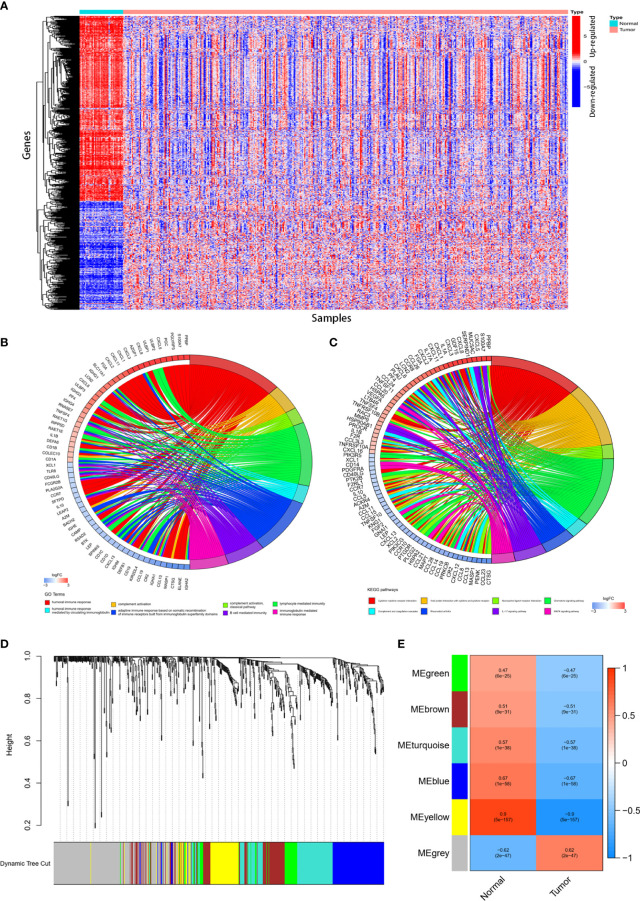
Identification of differentially expressed immune-related genes (DEIRGs) and construction of co-expression modules. 680DEIRGs were identified. **(A)** Heatmap of the 680 DEIRGs. The DEIRGs were enriched in GO terms **(B)** related with humoral immune response, complement activation, adaptive immune response, B cell mediated immunity, lymphocyte mediated immunity and immunoglobulin mediate immune response and KEGG pathways **(C)** related with cytokine-cytokine receptor interaction, complement and coagulation cascades, viral protein interaction with cytokine and cytokine receptor, IL-17 signaling pathway, chemokine signaling pathway, MAPK signal pathway. Six co-expression modules were constructed through WGCNA. The yellow module has the strongest correlation with colon cancer. **(D)** Dendrogram of the six modules. **(E)** Heatmap of the correlation between module eigengenes and sample types.

Gene expression in 39 normal tissues and 398 colon cancer tissues were compared and totally 680 IRGs were differentially expressed (**Figure 1A**). The differentially expressed IRGs (DEIRGs) were enriched in KEGG pathways related with cytokine-cytokine receptor interaction, complement and coagulation cascades, viral protein interaction with cytokine and cytokine receptor, IL-17 signaling pathway, chemokine signaling pathway, MAPK signal pathway. GO analysis showed that the differentially expressed IRGs were also enriched in GO terms related with humoral immune response, complement activation, adaptive immune response, B cell mediated immunity, lymphocyte mediated immunity and immunoglobulin mediate immune response (**Figures 1B, C**).

### Construction of co-expression modules and identification of key module

Totally 398 colon cancer samples were used to construct the co-expression modules using WGCNA R package. Based on the 680 DEIRGs, six co-expression modules were constructed (**Figure 1D**). The yellow module (with a correlation coefficient of 0.9, and p value= 5× 10^‐157^) has the strongest correlation with colon cancer (**Figure 1E**). Therefore, the yellow module was selected for further analysis.

### Construction and validation of immune-related prognostic index model

**Figure 2:**
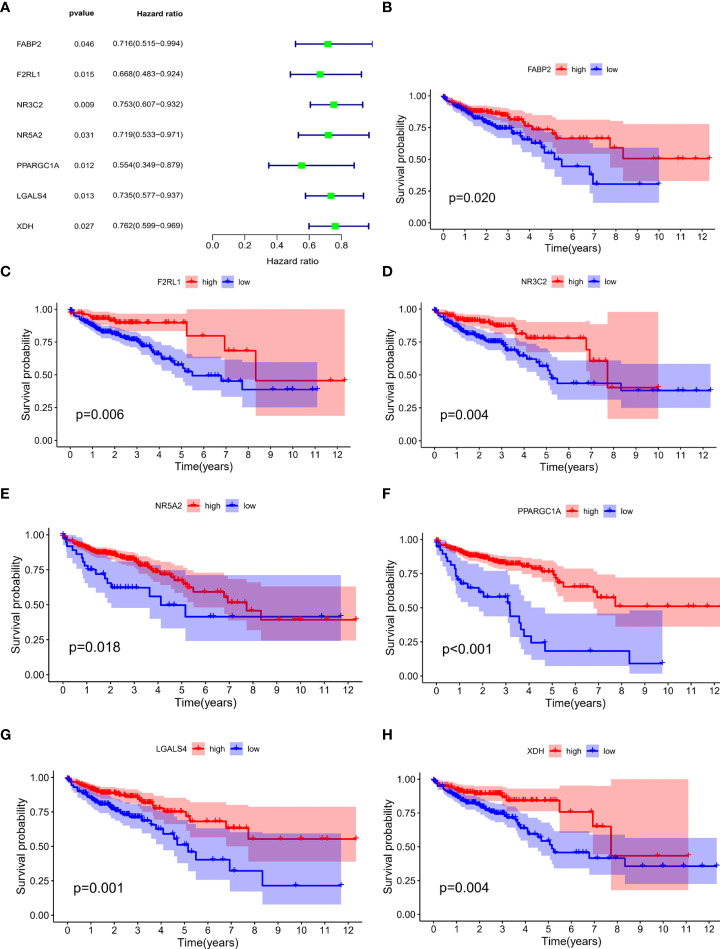
Univariate cox regression analysis was performed to identify survival-related IRGs. **(A)** FABP2, F2RL1, NR3C2, NR5A2, PPARGC1A, LGALS4 and XDH were identified as survival-related genes. Patients with high FABP2 **(B)**, F2RL1 **(C)**, NR3C2 **(D)**, NR5A2 **(E)**, PPARGC1A **(F)**, LGALS4 **(G)** and XDH **(H)** expression had better survival than patients with low expression of those IRGs.

There were 76 IRGs (**Supplementary Material S2**) included in the yellow module and 73 genes were shared in TCGA-COAD and GEO datasets. Univariate cox analysis was performed to identify survival-related IRGs. FABP2, F2RL1, NR3C2, NR5A2, PPARGC1A, LGALS4 and XDH were identified as survival-related genes (**Figures 2A–H**). Multivariate cox regression analysis showed that three IRGs (NR5A2, PPARGC1A and LGALS4) were independent prognostic IRGs. Thus, NR5A2, PPARGC1A and LGALS4 were used to establish the IRGPI model using the multivariate cox proportional hazards models. The IRGPI was calculated as follow:


IRGPI=−0.290×expression (NR5A2)−0.480×expression(PPARGC1A)−0.236×expression (LGALS4)

**Figure 3:**
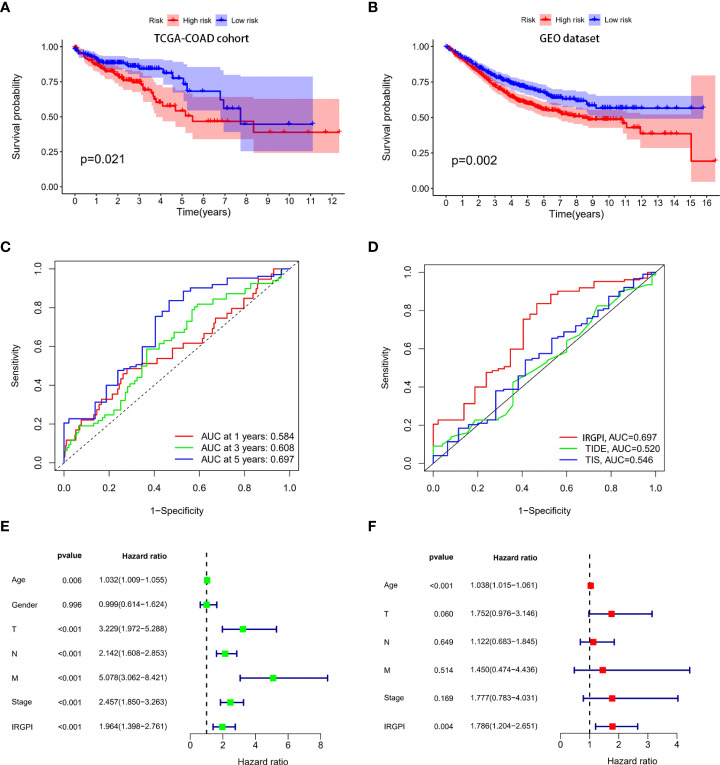
Construction and validation of immune-related prognostic index (IRGPI) model. IRGPI model was constructed using the multivariate cox proportional hazards model. Patients in the TCGA-COAD cohort were used as training set and patients in the GEO cohort were used as the validation set. ROC curves were plotted and AUCs were calculated to estimate the power of the IRGPI model to predict prognosis. **(A)** Survival of colon patients in high-risk and low risk groups in the TCGA-COAD cohort. Patients in the high risk group showed worse survival than patients in the low risk group. **(B)** Survival of colon patients in high-risk and low risk group in the GEO cohort. Patients in the high risk group showed worse survival than patients in the low risk group. **(C)** ROC curves of the IRGPI model for 1-year, 3-year and 5-year. The AUCs of ROC for 1-year, 3-year and 5-year were 0.584,0.608 and 0.697, respectively. **(D)** ROC curves of IRGPI model, TIS model and TIDE model for 5-years. The IRGPI model showed a stronger predictive power than the TIS and TIDE model. **(E)** Univariate cox regression analysis showed that IRGPI was correlated to survival. **(F)** Multivariate cox regression analysis showed that IRGPI was an independent prognostic factor for survival.

The risk scores of all the patients were calculated using the IRGPI model (**Supplementary Material S3**). Patients in the TCGA-COAD cohort were used as training set. IRGPI of patients in the training set was calculated and patients were classified into high-risk and low-risk group according to median values of IRGPI (median IRGPI=0.9990). Survival of patients in high-risk and low-risk group was compared. As indicated in **Figure 3A**, patients in high-risk group showed worse survival than patients in the low-risk group. Patients in GEO datasets (GSE17536, GSE17537, GSE29621, GSE40967) were used to validate the IRGPI model. IRGPI of patients in the validation set were calculated and patients were classified into high-risk and low-risk group based on the median value of IRGPI (media IRGPI=0.9990) in the training set. Results showed that high-risk group patients had worse outcome (**Figure 3B**). ROC curves were plotted and AUCs were calculated to estimate the power of the IRGPI model to predict prognosis. The AUC of the IRGPI model for 1-year, 3-year and 5-year were 0.584, 0.608 and 0.697, respectively (**Figure 3C**). The results indicated that the IRGPI model worked well at predicting long-term survival. The AUCs of the tumor inflammation signature (TIS) and tumor immune dysfunction and exclusion (TIDE) models were 0.546 and 0.520, respectively(**Figure 3D**). The AUCs of IRGPI model was larger than the TIS and TIDE model, which suggested that the IRGPI model in the current study showed a stronger predictive power for clinical outcomes. Univariate analysis showed that age, T stage, N stage, M stage, clinical stage and IRGPI were correlated with survival (**Figure 3E** and **Supplementary Material S4**). Multivariate cox regression analysis indicated that age and IRGPI were independent prognostic factors for survival (**Figure 3F** and **Supplementary Material S4**).

### Expression of IRGs in colon cancer cell lines

To estimate the expression level of IRGs used to construct the IRGPI in colon cancer cell lines, qRT-PCR was performed. We found that NR5A2, PPARGC1A and LGALS4 were all down-regulated in colon cancer cell lines, in comparison with normal colon epithelial cell line (**Supplementary Material S5**).

### Correlation of IRGPI with clinical features

**Figure 4:**
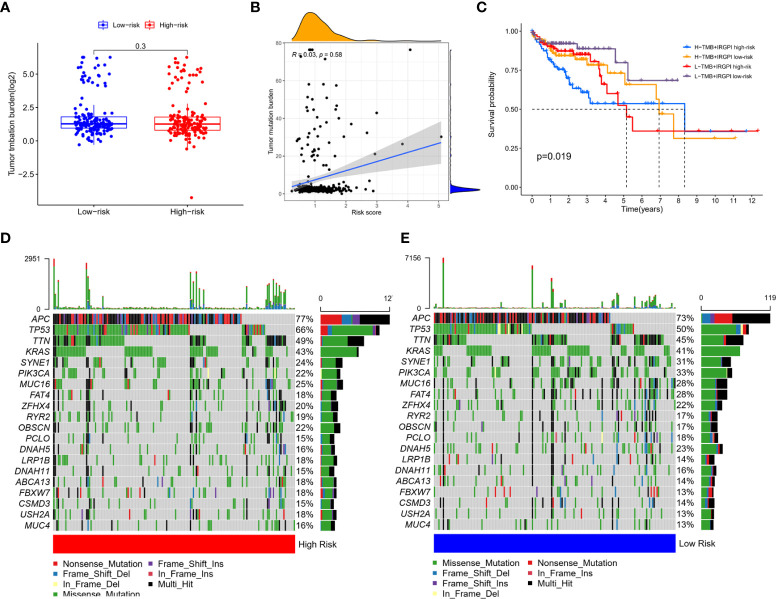
Correlation of IRGPI with clinical features. **(A)** TMB in high-risk and low-risk group showed no statistical difference. **(B)** Spearman correlation analysis of the correlation between IRGPI and TMB showed that the prognostic index was not correlated with TMB. **(C)** Survival of high -risk and low-risk group patients with different TMB levels. Patients with IRGPI low-risk and low TMB had the best survival. The 20 genes with the highest mutation frequency in high-risk **(D)** and low-risk **(E)** group. High-risk group had higher mutation frequency of APC, TP53, TTN, OBSCN and lower mutation frequency of SYNE1, PIK3CA and FAT4.

Age, gender, T stage, N stage, M stage and clinical stage of patients in high-risk group and low-risk group were compared. The results showed that there was no significant difference in the above clinical characteristics between the two groups (**Supplementary Materials S6**, **S7**). TMB in the two group patients was also compared. As shown in **Figure 4A**, TMB between two group patients had no significant difference. Spearman correlation analysis showed that the prognostic index was not correlated with TMB (**Figure 4B**). There were a few outliers with very high risk score (risk score>3). We excluded these outliers and performed the Spearman correlation analysis again, and the result remained consistent that IGRPI was not correlated with TMB (**Supplementary Material S8**). It was indicated that TMB was correlated with survival (14). To assess whether TMB combined with IRGPI can better predict survival outcome, we performed a stratified survival analysis. Results showed that patients with IRGPI low-risk and low TMB had the best survival (**Figure 4C**). Although TMB was not related to IRGPI, the distribution of somatic mutation between two groups showed some difference. The 20 genes with the highest mutation frequency were shown in **Figures 4D**, **E**. High-risk group had higher mutation frequency of APC, TP53, TTN, OBSCN and lower mutation frequency of SYNE1, PIK3CA and FAT4.

### Correlation of IRGPI with immune features

**Figure 5:**
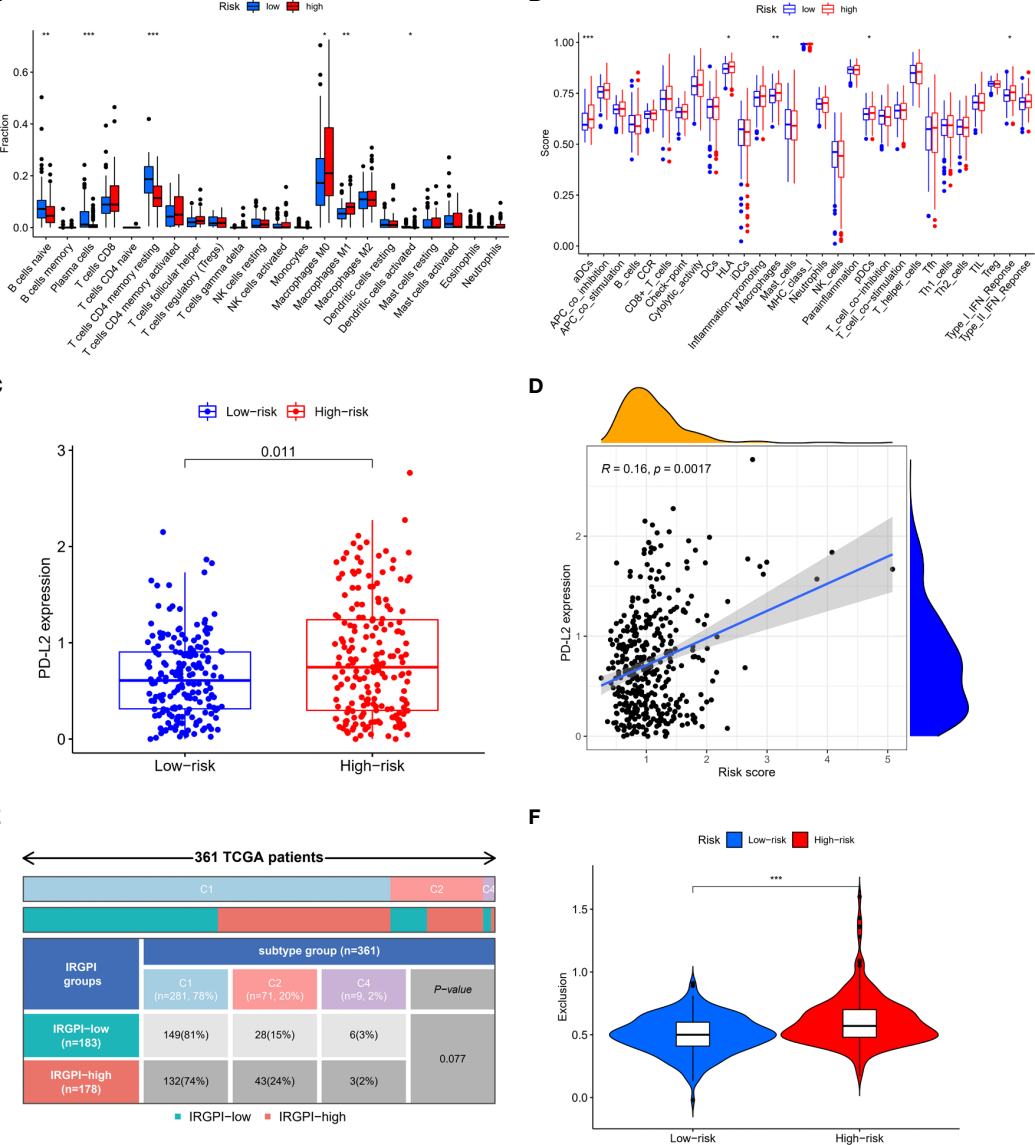
Correlation of IRGPI with immune features. **(A)** Immune cell infiltrations in high-risk and low-risk patients. High-risk group patients had higher infiltration level of M0 macrophages, M1 macrophages and lower infiltration level of naïve B cells, plasma cells and resting CD4+ memory T cells. **(B)** Immune function score in high-risk and low-risk patients. High-risk group had higher aDCs, HLA, macrophages, pDCs and type I IFN-response. **(C)** PD-L2 expression in high-risk and low-risk patients. PD-L2 expression was higher in high-risk group **(D)** Spearman correlation analysis of the correlation between PD-L2 and IRGPI. PD-L2 expression was positively related to IRGPI **(E)** Immune subtypes in high-risk and low-risk patients. No significant difference was observed in the proportion of each immune subtype between the high-risk and low-risk groups. **(F)** Immune exclusion score in high-risk and low-risk patients. Immune exclusion score was higher in high-risk group. *p<0.05; **p<0.01; ***p<0.001.

Immune cell infiltrations in high-risk and low-risk patients were compared. High-risk group patients had higher infiltration level of M0 macrophages, M1 macrophages and lower infiltration level of naïve B cells, plasma cells and resting CD4+ memory T cells (**Figure 5A**). Immune function score was analyzed and results showed that high-risk group had higher aDCs, HLA, macrophages, pDCs and type I IFN-response (**Figure 5B**). Immune checkpoint expression levels in the two groups were analyzed. There was no difference in the expression of PD-L1 and CTLA4 between the two groups (**Supplementary Material S9**). However, PD-L2 expression was higher in high-risk group and was positively related to IRGPI (**Figure 5C, D**). To checked whether the results were affected by a small bunch of outliers with very high risk score (risk score>3), we re-analyzed the correlation after excluded the outliers, and the results showed that risk score remained not correlated with PD-L1 and CTLA4, but remained correlated with PD-L2 (**Supplementary Material S10**). Three immune subtypes were identified in colon patients. However, no significant difference was observed in the proportion of each immune subtype between the high-risk and low-risk groups, though a trend of difference was shown (**Figure 5E**). We further compared the immune dysfunction and immune exclusion score in the two group patients. Immune dysfunction score did not show significant difference (**Supplementary Material S9**) while immune exclusion score was elevated in high-risk group (**Figure 5F**).

### Screening of pathways related to high-risk and low-risk phenotype

**Figure 6:**
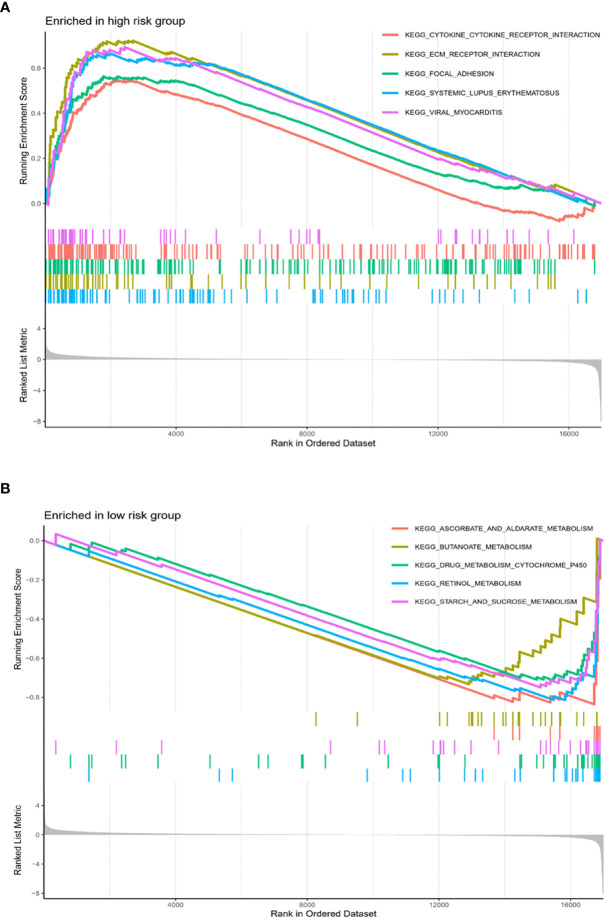
Pathways related to high-risk and low-risk phenotype. Pathways related to Cytokine-cytokine receptor interaction, ECM receptor interaction, focal adhesion were enriched in high-risk phenotype **(A)**. Pathways correlated with ascorbate and aldarate metabolism, butanoate metabolism, drug metabolism cytochrome p450, retinol metabolism and starch and sucrose metabolism were enriched in low-risk phenotype **(B)**.

Gene set enrichment analysis (GSEA) was performed to screen the pathways related to high-risk and low-risk phenotype. Pathways related to Cytokine-cytokine receptor interaction, ECM receptor interaction, focal adhesion were enriched in high-risk phenotype. Pathways correlated with ascorbate and aldarate metabolism, butanoate metabolism, drug metabolism cytochrome p450, retinol metabolism and starch and sucrose metabolism were enriched in low-risk phenotype (**Figures 6A, B**).

## Discussion

Immunotherapy has become an important part of colon cancer treatment. However, only a small percentage of patients, usually those with mismatch-repair-deficient (dMMR) or high microsatellite instability (MSI-H), benefit from immunotherapy (32). Identify of new biomarkers for response to immunotherapy and clinical outcomes may help to make better clinical decisions

An immune-related genes signature was reported in colorectal cancer. The IRG signature was constructed based on eight immune-related genes, including SLC10A2, FGF2, CCL28, NDRG1, ESM1, UCN, UTS2 and TRDC. The predictive power of the IRG signature for 5-year survival in colorectal cancer was 76.6% (33). Another study established a IRG signature in KRAS-mutant colorectal cancer based on four IRGs. This four IRGs signature was proved to be associated with immunosuppressive pathways and immune cell infiltration (32). However, the above two IRG prognostic models were constructed for colorectal cancer, and there was no IRG prognostic model specially constructed for colon cancer so far. Colon cancer is different from rectal cancer in anatomical location, embryonic origin, metastasis pattern, gene mutation profiles, etc (34). The treatment strategy and prognosis between colon cancer and rectal cancer were also different. For example, neoadjuvant chemoradiotherapy is suitable for local advanced rectal cancer, but not for colon cancer (34). Prognostic model specially established for colon cancer is essential.

In the current study, we defined the immune-related genes prognostic index (IRGPI) model specially for colon cancer for the first time. Firstly, we analyzed the immune landscape and identified the survival-related aberrant immune-related genes in colon cancer. Then three survival-related immune-related genes, NR5A2, PPARGC1A and LGALS4, were used to constructe an immune-related gene prognostic index (IRGPI) model and the prognosis predictive power of the model was validated in independent dataset. The coefficients of the three IRGs were all negative, which means increased expression of the three IRGs would decrease the risk and related to better clinical outcomes. NR5A2 is an orphan nuclear receptor and takes part in the regulation of metabolic progresses, cell differentiation and proliferation (35). Inhibition of NR5A2 decreased the production of pro-inflammatory cytokine and impaired T cell function (36, 37). PPARGC1A, also known as PGC-1α, is a transcriptional coactivator and regulates the transcription of many genes related cellular metabolism (38). Repression of PPARGC1A expression led to T cell dysfunction (39). LGALS4, coding a β-galactoside-binding protein call galectin-4, was commonly expressed in gastrointestinal tract. It regulates immune function by activating monocytes (40). It was reported that downregulation of LGALS4 was related to poor clinical outcome in colon cancer (41). It this study, we found the consistent results by showing that NR5A2, PPARGC1A and LGALS4 were down-regulated in colon cancer cell lines and downregulation of the three genes were related to poor prognosis. The results were also consistent with data from Gene-Cloud of Biotechnology Information (GCBI) database (https://www.gcbi.com.cn/gclib/html/index), which shows that NR5A2, PPARGC1A and LGALS4 were down-regulated in colon cancer (**Supplementary Material S11**).

We estimated the power for predicting clinical outcomes of the IRGPI by ROCs. The AUCs of ROC for 1-year, 3-year and 5-year were 0.584,0.608 and 0.697, which suggested that the IRGPI model has stronger power to predict long-term survival, especially for 5-year survival. It was reported that Tumor Immune Dysfunction and Exclusion (TIDE) model and Tumor Inflammation Signature (TIS) were correlated with survival outcome of cancer patients (30). Thus, we compared the IRGPI model with TIDE and TIS model using the ROCs and AUCs. Results showed that the IRPGI model had larger AUCs than the TIDE and TIS models, which suggested that IRPGI had a stronger predictive power for survival in colon cancer. The above results suggested that the IRGPI model constructed in this study may be helpful to predict the long-term survival of colon cancer patients.

We also further analyzed the correlation of the IRGPI model with clinical and immune features. TMB was a biomarker for response to immunotherapy and was related to survival in colon cancer (14). The current study showed that IRGPI was independent on TMB, but the combination of IRGPI and TMB can better distinguish patients with different prognosis. The results suggested that IRGPI and TMB may evaluate tumor immunity from different aspects. High-risk group patients had decreased naïve B cells, plasma cells, CD4+ memory T cells and increased M0 and M1 macrophage. B cells that have not been exposed to antigens are called naïve B cells (42). B cells activate T cells through antigen presentation and play an anti-tumor role. However, the function of naïve B cell in remains unknown (43). CD4+ T cells and M1 macrophage were correlated with increased anti-tumor response (14). In this study, high-risk patients, who have higher M1 cell infiltration, showed worse prognosis. One of the reasons maybe that other immune factors may have influence on survival, such as other immune cells, inflammatory factors, cytokines and metabolism products. Plasmacytoid DC (pDCs) promotes type I IFN secretion when stimulated by acute infection. It was described to be related to poor prognosis in cancer (44). Defect in HLA class I antigen processing machinery was associated with tumor development and was related to poor prognosis in several types of cancers (45). We showed that HLA, dendritic cells (DCs), macrophages and type I IFN response was elevated in high-risk group, which was consistent with previous reports. We analyzed the immune subtypes of the patients and found that most of the colon cancer patients were C1 or C2 subtypes. In comparison with high-risk group, low-risk group seemed to had high proportion of C1 immune subtype. Though the difference did not reach a statistical significance, it did show a trend. The result was consistent with previous report (32). Though immune dysfunction score in the two groups did not show statistical difference, high-risk group had a higher immune exclusion score. That maybe one of the reasons why high-risk patients showed worse survival outcome.

The IRGPI may help to predict survival and response to immunotherapy in colon cancer to some extent. The IRGPI model was the first prognostic model specially constructed for colon cancer patients and the IRGPI was validated by independent datasets. The predictive power of the IRGPI was stronger than other models, such as TIDE and TIS. The correlation of the IRGPI with clinical and immune characteristics were analyzed so that we can better understand the IRGPI model in various aspects. However, some limitations should be noticed. Firstly, patient’s treatment information was not taken into account when constructing the prognostic model. Secondly, the IRGPI should be further validated clinically. Thirdly, *in vitro* and *in vivo* experiments should be performed to better understanding the biological role of the IRGPI.

In conclusion, we constructed and validated an immune-related genes prognostic index (IRGPI) in colon cancer patients for the first time, and the IRGPI maybe a potential biomarker for prognosis and therapy.

## Data availability statement

The original contributions presented in the study are included in the article/**Supplementary Material**. Further inquiries can be directed to the corresponding author.

## Author contributions

YJ, JD, BL, YZ and SY had full access to all the data in the study and take responsibility for the integrity of the data and the accuracy of the data analysis. YJ, JD, BL, YZ and SY designed the study. Acquisition of data: YJ and JD. Analysis and interpretation of data: YJ, JD and SY. Statistical analysis: YJ, JD, BL, YZ and SY. Drafting and revising of the manuscript: YJ, JD and SY. All authors contributed to the article and approved the submitted version.

## Conflict of interest

The authors declare that the research was conducted in the absence of any commercial or financial relationships that could be construed as a potential conflict of interest.

## Publisher’s note

All claims expressed in this article are solely those of the authors and do not necessarily represent those of their affiliated organizations, or those of the publisher, the editors and the reviewers. Any product that may be evaluated in this article, or claim that may be made by its manufacturer, is not guaranteed or endorsed by the publisher.

## Supplementary material

Supplementary Material S1 Workflow chart of the study.

Supplementary Material S2 The 76 IRGs included in the yellow module.

Supplementary Material S3 The risk scores of patients in TCGA-COAD and GEO cohorts.

Supplementary Material S4 Univariate and multivariate cox regression analysis of the correlation between age, T stage, N stage, M stage, clinical stage andIRGPI and survival.

Supplementary Material S5 NR5A2, PPARGC1A and LGALS4 expression in normal colon epithelia cell line and colon cancer cell lines. NR5A2, PPARGC1A and LGALS4 expression were significantly decreased in colon cancer cell lines. (* P<0.05)

Supplementary Material S6 Age, gender, T stage, N stage, M stage and clinical stage of patients in high-risk group and low-risk group. There was no significant difference in the above clinical characteristics between the two groups.

Supplementary Material S7 Correlation of IRGPI with clinical features. There was no significant difference in the **(A)** Age **(B)** Gender **(C)** T stage **(D)** N stage **(E)** M stage **(F)** Clinical stage between the high risk and low risk groups.

Supplementary Material S8 We excluded these outliers and performed the Spearman correlation analysis again, and the result remained consistent that IGRPI was not correlated with TMB.

Supplementary Material S9 Immune checkpoint expression and immune dysfunction score in high-risk and low-risk group. Expression of PD-L1 and CTLA4 between the two groups showed no significant difference. Expression of PD-L1 and CTLA4 was not correlated with IRGPI. **(A)** CTLA4 expression. **(B)** Spearman correlation analysis of the correlation between CTLA4 and IRGPI. **(C)** PD-L1 expression. **(D)** Spearman correlation analysis of the correlation between PD-L1 and IRGPI. **(E)** Immune dysfunction score in high-risk and low-risk patients showed no statistical difference.

Supplementary Material S10 We re-analyzed the correlation after excluded the outliers, and the results showed that risk score remained not correlated with PD-L1 and CTLA4, but remained correlated with PD-L2.

Supplementary Material S11 NR5A2, PPARGC1A and LGALS4 were down-regulated in colon cancer. **(A)** LGALS4. **(B)** NR5A2. **(C)** PPARGC1A.
