# Supplementary figures and images for: Construction and validation of an immune-related genes prognostic index (IRGPI) model in colon cancer
Source: Front Endocrinol (Lausanne). 2022 Nov 9;13:963382. doi: 10.3389/fendo.2022.963382 (PMC9682206; doi:10.3389/fendo.2022.963382)

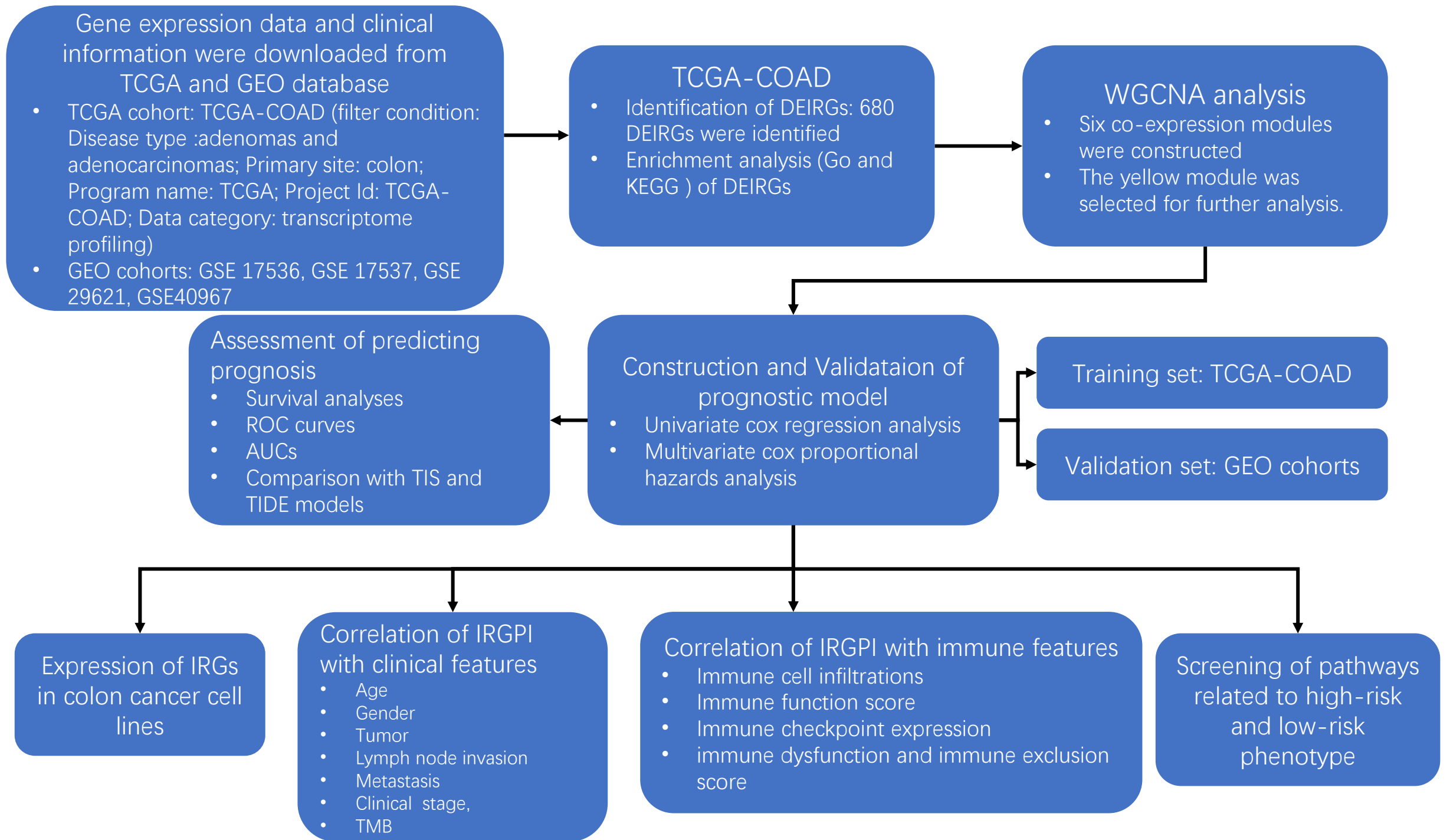

Supplement: Supplementary Material S1 — Workflow chart of the study. [file DataSheet_1.pdf]

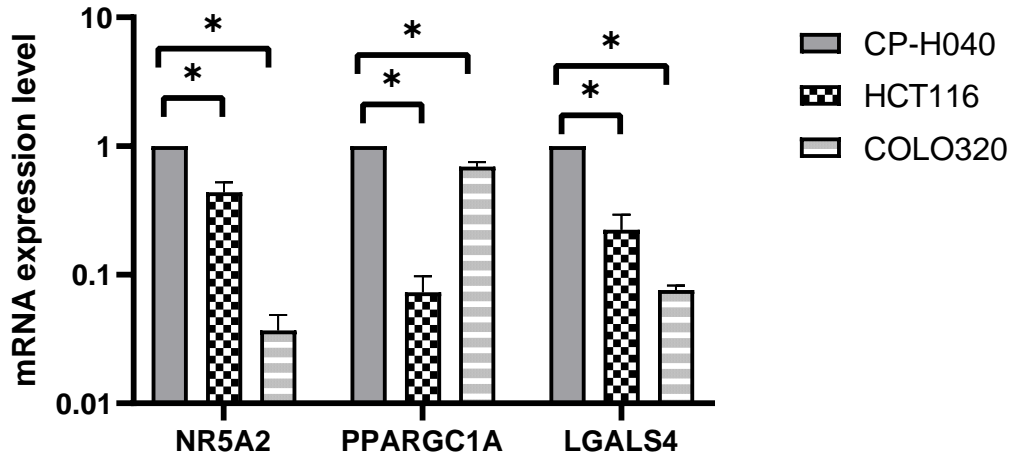

Supplement: Supplementary Material S5 — NR5A2, PPARGC1A and LGALS4 expression in normal colon epithelia cell line and colon cancer cell lines. NR5A2, PPARGC1A and LGALS4 expression were significantly decreased in colon cancer cell lines. (* P<0.05) [file DataSheet_5.pdf]

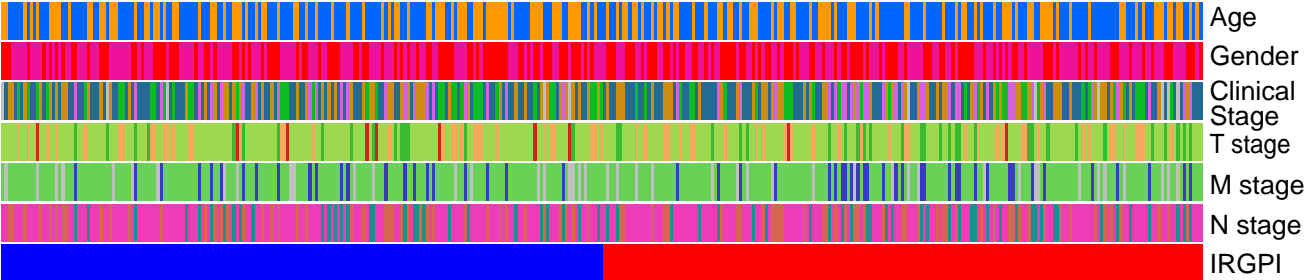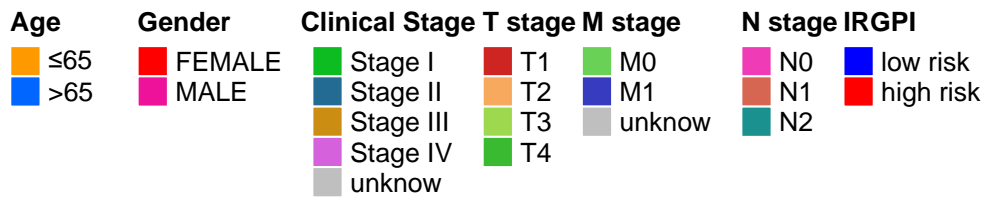

Supplement: Supplementary Material S6 — Age, gender, T stage, N stage, M stage and clinical stage of patients in high-risk group and low-risk group. There was no significant difference in the above clinical characteristics between the two groups. [file DataSheet_6.pdf]

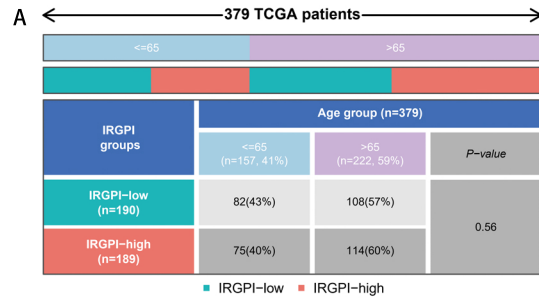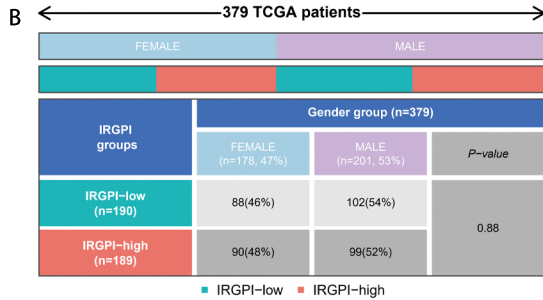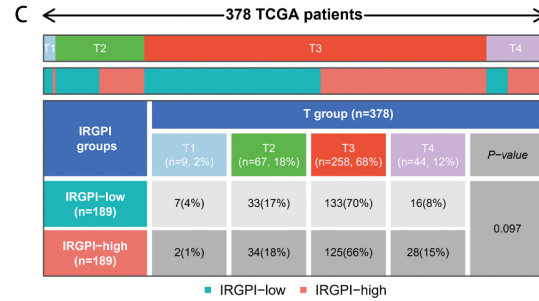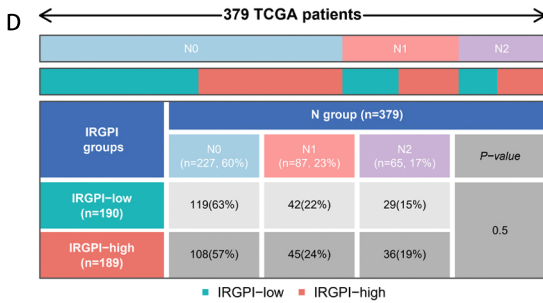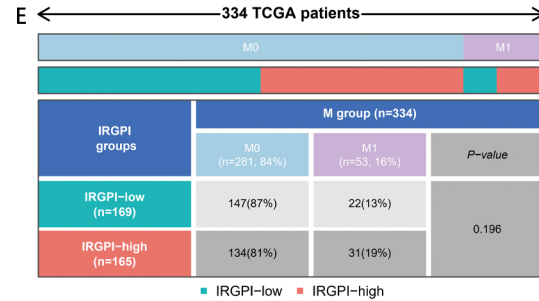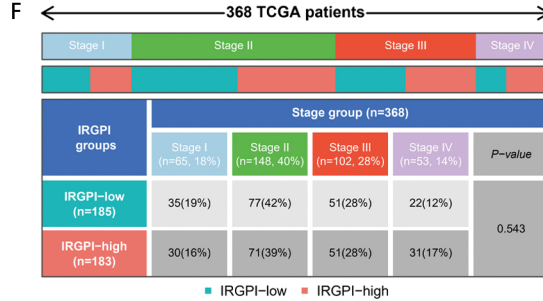

Supplement: Supplementary Material S7 — Correlation of IRGPI with clinical features. There was no significant difference in the (A) Age (B) Gender (C) T stage (D) N stage (E) M stage (F) Clinical stage between the high risk and low risk groups. [file DataSheet_7.pdf]

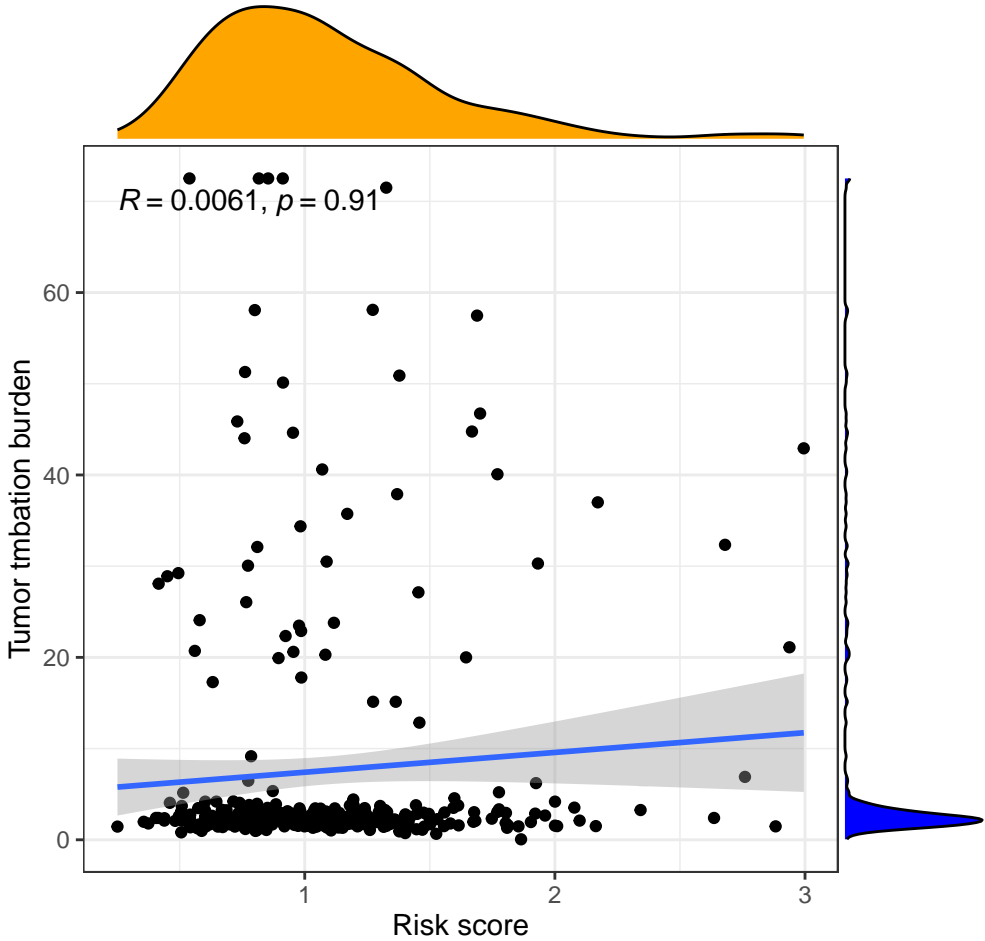

Supplement: Supplementary Material S8 — We excluded these outliers and performed the Spearman correlation analysis again, and the result remained consistent that IGRPI was not correlated with TMB. [file DataSheet_8.pdf]

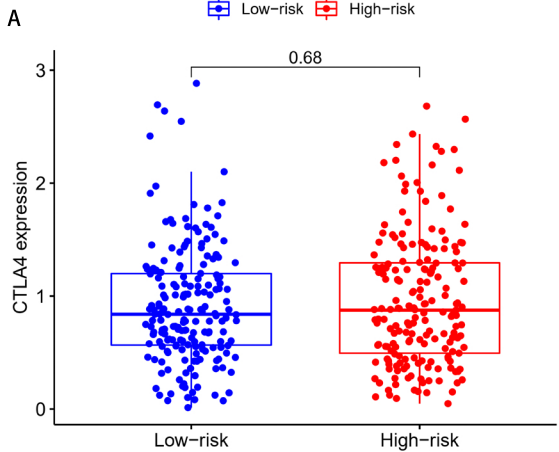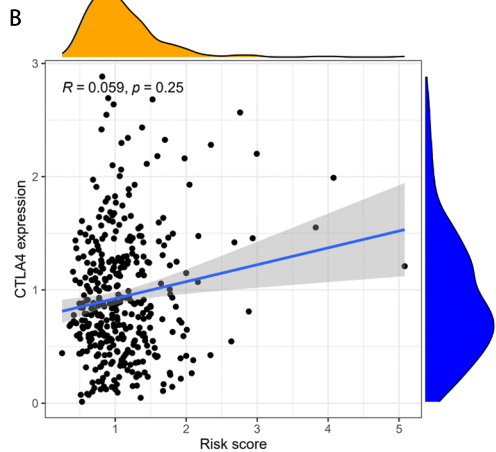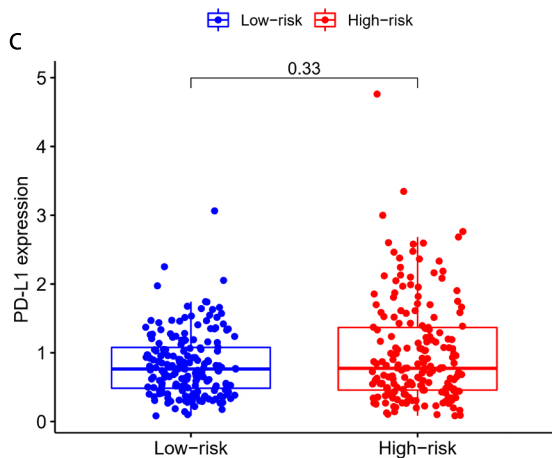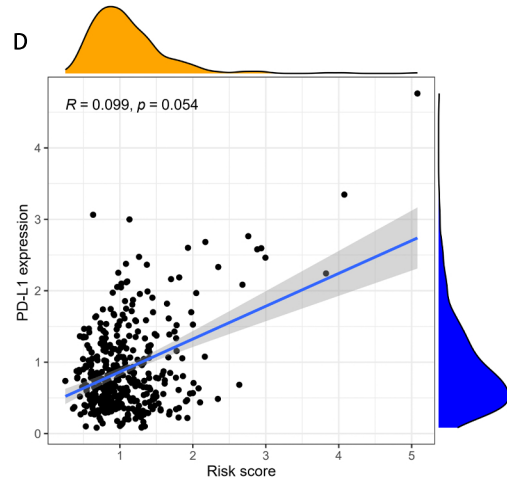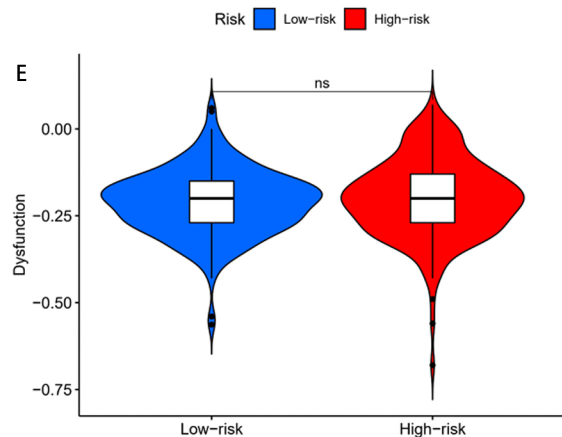

Supplement: Supplementary Material S9 — Immune checkpoint expression and immune dysfunction score in high-risk and low-risk group. Expression of PD-L1 and CTLA4 between the two groups showed no significant difference. Expression of PD-L1 and CTLA4 was not correlated with IRGPI. (A) CTLA4 expression. (B) Spearman correlation analysis of the correlation between CTLA4 and IRGPI. (C) PD-L1 expression. (D) Spearman correlation analysis of the correlation between PD-L1 and IRGPI. (E) Immune dysfunction score in high-risk and low-risk patients showed no statistical difference. [file DataSheet_9.pdf]

**A**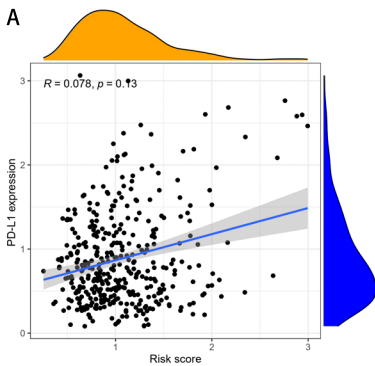**B**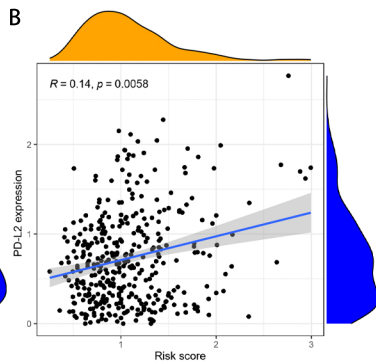**C**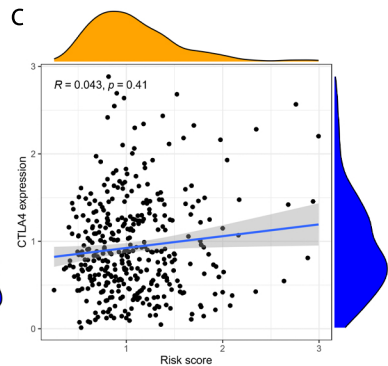

Supplement: Supplementary Material S10 — We re-analyzed the correlation after excluded the outliers, and the results showed that risk score remained not correlated with PD-L1 and CTLA4, but remained correlated with PD-L2. [file DataSheet_10.pdf]

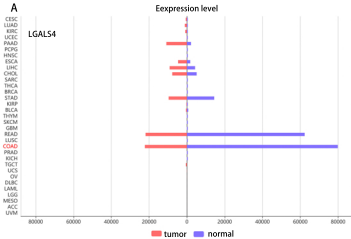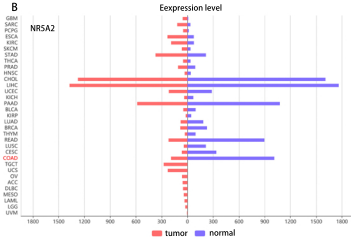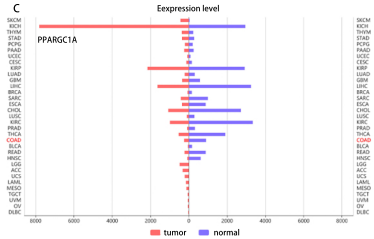

Supplement: Supplementary Material S11 — NR5A2, PPARGC1A and LGALS4 were down-regulated in colon cancer. (A) LGALS4. (B) NR5A2. (C) PPARGC1A. [file DataSheet_11.pdf]
