# Supplementary material for: Construction and validation of an immune-related genes prognostic index (IRGPI) model in colon cancer
Source: Front Endocrinol (Lausanne). 2022 Nov 9;13:963382. doi: 10.3389/fendo.2022.963382 (PMC9682206; doi:10.3389/fendo.2022.963382)
Supplement: Supplementary Material S2 — The 76 IRGs included in the yellow module. [file DataSheet_2.pdf]

CTSS
HSP90AB1
SLC10A2
PSMD14
DEFB1
LEAP2
S100A11
ZC3HAV1L
FABP2
OASL
AEN
TLR3
TNFSF10
RNASEL
F2RL1
CCL28
APOBEC3A
NDRG1
HMOX1
RSAD2
CCL15
ACKR2
CCL15-CCL14
PTK2B
CHP1
CHP2
EDN2
EDN3
SEMA6A
SEMA6D
PTGDR2
PLXNA1
PLXNA2
BMP2
BMP3
BMP5
FAM3D
GUCA2A
NTS
SECTM1
ACVR1C
ACVRL1
CNTFR
FGFR2

HTR3C
HTR3E
IL6R
LGR4
NR1H4
NR3C2
NR5A2
PTGER4
SSTR1
TNFRSF12A
VIPR1
FAS
CDK4
LGALS3
PPARGC1A
USP2
RIPK2
RNF125
TRIM36
TRIM28
CASP7
RHBDF2
APOBEC3B
PPARGC1B
HSPD1
DDX21
RCAN1
LGALS2
CEACAM1
LGALS4
XDH
BDKRB2
BMX
