# Supplementary material for: Construction and validation of an immune-related genes prognostic index (IRGPI) model in colon cancer
Source: Front Endocrinol (Lausanne). 2022 Nov 9;13:963382. doi: 10.3389/fendo.2022.963382 (PMC9682206; doi:10.3389/fendo.2022.963382)
Supplement: Supplementary Material S3 — The risk scores of patients in TCGA-COAD and GEO cohorts. [file DataSheet_3.pdf]

TCGA			GEO		
id	riskScore	Risk	id	riskScore	Risk
TCGA-AA-3867	0.788898	low	GSM972361	6.422413	high
TCGA-CA-6719	1.186345	high	GSM972275	6.373475	high
TCGA-NH-A50V	0.804965	low	GSM437133	4.872381	high
TCGA-AA-A01C	1.135858	high	GSM972008	4.018156	high
TCGA-AA-A00O	0.864662	low	GSM437204	4.001671	high
TCGA-AZ-4615	1.644963	high	GSM437307	3.758792	high
TCGA-AA-A01V	0.876142	low	GSM972213	3.738165	high
TCGA-AA-A00D	1.977399	high	GSM972470	3.278068	high
TCGA-A6-5657	1.864769	high	GSM437118	3.222239	high
TCGA-AA-3522	0.547827	low	GSM972499	3.21686	high
TCGA-D5-6923	1.768035	high	GSM972466	3.105034	high
TCGA-DM-A28A	1.044306	high	GSM734145	3.064835	high
TCGA-AA-3848	1.449448	high	GSM972334	2.955945	high
TCGA-AA-3667	2.000362	high	GSM972026	2.799025	high
TCGA-F4-6459	0.54213	low	GSM972059	2.726464	high
TCGA-DM-A1D0	1.033543	high	GSM972341	2.608232	high
TCGA-AA-3517	1.176276	high	GSM437254	2.605077	high
TCGA-D5-5541	1.404251	high	GSM972097	2.518543	high
TCGA-A6-2686	1.378455	high	GSM972039	2.506666	high
TCGA-AA-3518	0.662241	low	GSM972501	2.476106	high
TCGA-AA-3984	0.912307	low	GSM972452	2.433377	high
TCGA-AZ-6599	0.87227	low	GSM734148	2.431876	high
TCGA-D5-6922	0.685069	low	GSM437272	2.384885	high
TCGA-A6-3810	1.037447	high	GSM437301	2.306038	high
TCGA-AA-A01Q	1.01957	high	GSM972171	2.233725	high
TCGA-AA-3979	1.084937	high	GSM972353	2.212199	high
TCGA-A6-5667	1.032716	high	GSM972458	2.181941	high
TCGA-CM-6172	0.374863	low	GSM972433	2.162203	high
TCGA-AZ-6600	0.878093	low	GSM972343	2.128149	high
TCGA-AA-3971	0.578455	low	GSM971958	2.122594	high
TCGA-AA-3666	0.811682	low	GSM972194	2.121275	high
TCGA-D5-6898	0.622592	low	GSM972210	2.12126	high
TCGA-D5-6537	1.140064	high	GSM972209	2.119387	high
TCGA-SS-A7HO	1.594533	high	GSM437223	2.113608	high
TCGA-CK-5915	1.022519	high	GSM972068	2.107125	high
TCGA-AA-A00L	0.674576	low	GSM972002	2.095906	high
TCGA-G4-6321	0.50644	low	GSM437212	2.091286	high
TCGA-AA-3489	1.357136	high	GSM972072	2.069252	high
TCGA-AA-A01S	0.762754	low	GSM972354	2.067934	high
TCGA-D5-6931	0.774334	low	GSM972409	2.05435	high
TCGA-T9-A92H	0.777881	low	GSM972397	2.02782	high
TCGA-A6-5656	0.771607	low	GSM972294	2.022965	high
TCGA-AD-6965	1.263016	high	GSM972481	2.022526	high
TCGA-A6-6140	0.451379	low	GSM437148	2.015511	high
TCGA-CM-4744	1.496053	high	GSM972105	2.014663	high
TCGA-AD-A5EJ	0.983055	low	GSM971996	2.014617	high
TCGA-AA-3970	0.612337	low	GSM734128	2.006111	high
TCGA-CK-5914	1.329401	high	GSM972047	1.978347	high
TCGA-A6-5666	1.682123	high	GSM972476	1.949413	high
TCGA-4N-A93T	0.798957	low	GSM972011	1.927829	high
TCGA-CM-6171	0.450873	low	GSM971966	1.889975	high
TCGA-G4-6309	0.560662	low	GSM972479	1.882163	high
TCGA-AA-3697	0.809599	low	GSM971970	1.879537	high
TCGA-G4-6586	0.494779	low	GSM734170	1.866609	high
TCGA-A6-6650	0.608046	low	GSM972224	1.866093	high
TCGA-AZ-4616	1.261575	high	GSM437203	1.864667	high

TCGA-AA-A02J	1.305985	high
TCGA-D5-6538	1.296184	high
TCGA-NH-A6GB	1.30151	high
TCGA-A6-2682	1.260576	high
TCGA-AA-A02Y	0.497076	low
TCGA-AA-3850	0.618252	low
TCGA-CK-6747	0.602084	low
TCGA-A6-6782	1.117505	high
TCGA-CM-5860	1.120209	high
TCGA-AA-A00R	2.348085	high
TCGA-A6-6653	0.416082	low
TCGA-AD-6890	0.97892	low
TCGA-D5-6539	0.251421	low
TCGA-AD-A5EK	1.904147	high
TCGA-A6-3808	1.088059	high
TCGA-AA-3506	0.601312	low
TCGA-G4-6298	1.803455	high
TCGA-AZ-6608	1.321007	high
TCGA-AA-3678	1.043341	high
TCGA-A6-5665	0.73038	low
TCGA-G4-6306	0.629768	low
TCGA-F4-6806	0.625756	low
TCGA-AA-A01T	1.15867	high
TCGA-A6-6651	1.240647	high
TCGA-AA-3812	0.951795	low
TCGA-F4-6809	0.903975	low
TCGA-AA-A03J	0.913756	low
TCGA-AA-3815	1.082699	high
TCGA-CM-6169	1.178891	high
TCGA-F4-6855	1.002311	high
TCGA-G4-6294	0.851132	low
TCGA-A6-3807	1.477526	high
TCGA-AA-3846	1.056697	high
TCGA-F4-6703	2.759895	high
TCGA-AZ-4313	1.458442	high
TCGA-F4-6461	0.687584	low
TCGA-AA-3662	1.39123	high
TCGA-A6-2678	0.502811	low
TCGA-CM-6675	0.663754	low
TCGA-AY-4071	0.693458	low
TCGA-CA-5797	0.542539	low
TCGA-A6-5664	0.80657	low
TCGA-AA-A02H	0.973534	low
TCGA-AA-3862	0.801693	low
TCGA-CA-6716	1.240047	high
TCGA-A6-5661	0.773185	low
TCGA-D5-6535	0.508524	low
TCGA-A6-4105	1.063034	high
TCGA-G4-6307	0.720762	low
TCGA-A6-2680	1.003705	high
TCGA-CM-5862	1.287891	high
TCGA-DM-A28G	0.601747	low
TCGA-5M-AATE	1.421809	high
TCGA-DM-A28H	1.559008	high
TCGA-CM-4747	1.386718	high
TCGA-G4-6314	1.232252	high
TCGA-AA-3930	1.419933	high
TCGA-CA-6718	4.077011	high

GSM437142	1.860765	high
GSM972205	1.841343	high
GSM971981	1.839218	high
GSM972207	1.825241	high
GSM437286	1.816237	high
GSM972445	1.809431	high
GSM972181	1.804039	high
GSM972225	1.802094	high
GSM972360	1.79753	high
GSM972157	1.792792	high
GSM437213	1.785916	high
GSM972283	1.765784	high
GSM972123	1.76132	high
GSM971962	1.745174	high
GSM437315	1.740123	high
GSM972408	1.736956	high
GSM972232	1.728843	high
GSM437249	1.72745	high
GSM972015	1.725506	high
GSM971986	1.723063	high
GSM437284	1.718824	high
GSM437226	1.717681	high
GSM971963	1.708095	high
GSM972367	1.707455	high
GSM437124	1.705934	high
GSM972428	1.704463	high
GSM972113	1.699419	high
GSM437318	1.688703	high
GSM437256	1.686148	high
GSM437267	1.685503	high
GSM972419	1.684046	high
GSM972363	1.676457	high
GSM437175	1.675731	high
GSM734111	1.668597	high
GSM972276	1.668071	high
GSM972186	1.666371	high
GSM972271	1.649856	high
GSM972120	1.647835	high
GSM437173	1.641461	high
GSM437106	1.637879	high
GSM972349	1.633394	high
GSM437209	1.632648	high
GSM437189	1.632605	high
GSM437278	1.631615	high
GSM972162	1.629077	high
GSM972010	1.621801	high
GSM972126	1.621445	high
GSM972251	1.6198	high
GSM972122	1.618587	high
GSM972324	1.609537	high
GSM972208	1.60515	high
GSM972064	1.601483	high
GSM437239	1.600493	high
GSM972318	1.599509	high
GSM972066	1.597244	high
GSM972345	1.593121	high
GSM437196	1.583988	high
GSM972335	1.582881	high

TCGA-AD-6963	2.163671	high	GSM972217	1.582634	high
TCGA-AA-3982	0.601827	low	GSM734160	1.582049	high
TCGA-CM-6680	1.342021	high	GSM437236	1.575735	high
TCGA-AZ-6603	0.958613	low	GSM971973	1.569825	high
TCGA-A6-6142	1.582238	high	GSM972420	1.568306	high
TCGA-AZ-4315	0.853783	low	GSM972485	1.568232	high
TCGA-AD-6895	1.369393	high	GSM972262	1.562651	high
TCGA-A6-2672	0.977254	low	GSM972484	1.56106	high
TCGA-AA-3510	0.539222	low	GSM437242	1.557139	high
TCGA-AA-3527	1.343347	high	GSM972176	1.556583	high
TCGA-F4-6807	1.120384	high	GSM972220	1.554919	high
TCGA-G4-6310	1.35885	high	GSM972235	1.552875	high
TCGA-AA-A00K	0.718045	low	GSM972504	1.551978	high
TCGA-D5-6529	0.982289	low	GSM734122	1.551745	high
TCGA-AA-A01I	0.541336	low	GSM734162	1.550093	high
TCGA-AA-A01Z	1.326142	high	GSM972413	1.548297	high
TCGA-G4-6588	0.913146	low	GSM972069	1.545654	high
TCGA-CK-5912	1.556893	high	GSM437293	1.538113	high
TCGA-CM-6679	1.034733	high	GSM437152	1.537227	high
TCGA-AA-3519	0.983652	low	GSM972356	1.532155	high
TCGA-AZ-6607	2.882501	high	GSM972492	1.528824	high
TCGA-A6-5659	1.402216	high	GSM437241	1.522192	high
TCGA-CM-5344	0.918783	low	GSM972247	1.517024	high
TCGA-AA-3831	0.64769	low	GSM437198	1.515614	high
TCGA-DM-A0X9	0.714806	low	GSM437265	1.511407	high
TCGA-D5-6541	0.506458	low	GSM437188	1.511154	high
TCGA-A6-4107	0.756913	low	GSM972103	1.495972	high
TCGA-AA-3875	0.803526	low	GSM437117	1.491039	high
TCGA-G4-6295	0.931101	low	GSM437314	1.485191	high
TCGA-DM-A28F	0.514309	low	GSM972082	1.483642	high
TCGA-CM-4743	1.932185	high	GSM437240	1.479967	high
TCGA-F4-6808	0.953125	low	GSM972108	1.478456	high
TCGA-AA-A00U	1.160083	high	GSM972376	1.477305	high
TCGA-AA-A017	0.846545	low	GSM972462	1.474965	high
TCGA-AA-A01X	1.80886	high	GSM972313	1.474344	high
TCGA-CM-5861	0.810391	low	GSM972281	1.472657	high
TCGA-D5-5537	1.530204	high	GSM734131	1.472303	high
TCGA-CK-4948	1.366293	high	GSM972288	1.471591	high
TCGA-AA-3968	1.069637	high	GSM971968	1.470082	high
TCGA-D5-6530	1.4545	high	GSM437229	1.467616	high
TCGA-AA-3818	0.788636	low	GSM972079	1.464179	high
TCGA-G4-6315	0.678986	low	GSM734114	1.461312	high
TCGA-AA-3562	0.716393	low	GSM972095	1.458917	high
TCGA-AA-3872	1.401777	high	GSM437201	1.457605	high
TCGA-AA-3845	0.632223	low	GSM437130	1.457077	high
TCGA-AA-A010	0.81655	low	GSM972024	1.456936	high
TCGA-AA-3529	0.87858	low	GSM972286	1.454243	high
TCGA-A6-2675	0.754446	low	GSM972426	1.451121	high
TCGA-G4-6628	1.701091	high	GSM437245	1.4497	high
TCGA-AD-6964	2.680205	high	GSM437135	1.449638	high
TCGA-AD-6888	0.840999	low	GSM734151	1.449221	high
TCGA-AA-3973	1.319379	high	GSM734130	1.448724	high
TCGA-AA-3680	0.531929	low	GSM734123	1.447587	high
TCGA-DM-A1D8	2.342608	high	GSM437174	1.446353	high
TCGA-AM-5821	5.079493	high	GSM972131	1.444389	high
TCGA-CK-5916	1.170039	high	GSM437313	1.442395	high
TCGA-NH-A50T	0.883381	low	GSM971961	1.442036	high
TCGA-AA-A00Z	1.664288	high	GSM437116	1.44162	high

TCGA-CM-6674	0.923483	low
TCGA-AA-3833	0.785672	low
TCGA-AA-3841	1.326657	high
TCGA-D5-6532	0.705584	low
TCGA-DM-A0XD	2.077914	high
TCGA-AA-3492	0.761483	low
TCGA-AA-A022	2.171559	high
TCGA-AA-3844	0.785714	low
TCGA-AD-6901	1.776427	high
TCGA-AA-A01P	2.937813	high
TCGA-G4-6293	0.674153	low
TCGA-AA-3664	0.668849	low
TCGA-G4-6317	1.000366	high
TCGA-D5-6929	0.845567	low
TCGA-A6-6648	0.71642	low
TCGA-F4-6569	1.960841	high
TCGA-A6-2684	1.812852	high
TCGA-3L-AA1B	0.855526	low
TCGA-AA-A00A	0.552632	low
TCGA-CM-5349	1.277457	high
TCGA-AA-3534	1.032494	high
TCGA-G4-6303	1.218547	high
TCGA-QG-A5Z2	0.895364	low
TCGA-CA-5256	1.108558	high
TCGA-CM-5341	1.042195	high
TCGA-F4-6854	1.117161	high
TCGA-AA-3842	0.811593	low
TCGA-A6-A5ZU	1.212464	high
TCGA-AA-3870	1.854554	high
TCGA-AA-3511	0.615871	low
TCGA-CM-6167	1.615353	high
TCGA-CM-5348	1.180563	high
TCGA-AA-3514	1.296288	high
TCGA-QG-A5YW	0.982169	low
TCGA-CA-6715	1.674157	high
TCGA-NH-A6GA	1.004601	high
TCGA-AA-A004	1.315576	high
TCGA-AD-6889	0.759211	low
TCGA-D5-6536	0.687004	low
TCGA-AA-A029	1.024583	high
TCGA-AZ-5407	0.853952	low
TCGA-AA-3548	0.839317	low
TCGA-A6-6649	0.405327	low
TCGA-AA-3655	0.793859	low
TCGA-G4-6311	1.49125	high
TCGA-G4-6297	1.240142	high
TCGA-5M-AAT6	2.994974	high
TCGA-DM-A1DB	0.999043	high
TCGA-CM-6678	0.735226	low
TCGA-AA-3660	1.025738	high
TCGA-DM-A0XF	1.401712	high
TCGA-AY-A71X	0.64111	low
TCGA-DM-A28C	0.5322	low
TCGA-AA-3560	0.782078	low
TCGA-AA-3552	0.994045	low
TCGA-CM-6166	1.173567	high
TCGA-AZ-5403	0.756635	low
TCGA-G4-6299	1.613436	high

GSM437160	1.441269	high
GSM972170	1.432355	high
GSM972406	1.431857	high
GSM972467	1.430367	high
GSM972308	1.429318	high
GSM972284	1.424202	high
GSM972184	1.423473	high
GSM437153	1.422445	high
GSM972422	1.419902	high
GSM972401	1.41971	high
GSM437126	1.415961	high
GSM972143	1.415107	high
GSM972369	1.412835	high
GSM437102	1.411227	high
GSM734118	1.409308	high
GSM972389	1.408692	high
GSM972179	1.40794	high
GSM972451	1.405256	high
GSM972051	1.39878	high
GSM437270	1.397262	high
GSM972430	1.389169	high
GSM972061	1.38816	high
GSM971991	1.388091	high
GSM437300	1.38629	high
GSM972395	1.384145	high
GSM734133	1.383288	high
GSM437179	1.383079	high
GSM972104	1.382642	high
GSM972112	1.379031	high
GSM972193	1.37738	high
GSM972216	1.373794	high
GSM972491	1.373657	high
GSM972380	1.3707	high
GSM437190	1.370569	high
GSM972464	1.367912	high
GSM437145	1.365868	high
GSM437112	1.365504	high
GSM437287	1.36457	high
GSM972365	1.364026	high
GSM972519	1.361617	high
GSM972057	1.359352	high
GSM972502	1.357482	high
GSM437276	1.354611	high
GSM437207	1.354333	high
GSM437290	1.353919	high
GSM972425	1.353072	high
GSM972322	1.349576	high
GSM972314	1.346254	high
GSM971997	1.345877	high
GSM437319	1.345486	high
GSM972076	1.345231	high
GSM437280	1.343976	high
GSM972121	1.337959	high
GSM972004	1.337477	high
GSM972265	1.334374	high
GSM972089	1.333756	high
GSM972067	1.332577	high
GSM972410	1.331347	high

TCGA-AA-3496	1.74785	high	GSM437163	1.330217	high
TCGA-G4-6304	1.36395	high	GSM972054	1.327794	high
TCGA-CM-5864	0.885003	low	GSM972236	1.325395	high
TCGA-AY-A8YK	0.671905	low	GSM972212	1.325086	high
TCGA-CK-5913	0.954708	low	GSM972180	1.324183	high
TCGA-AZ-6605	1.00837	high	GSM437186	1.321812	high
TCGA-CM-4751	1.008848	high	GSM972018	1.31887	high
TCGA-CM-6677	1.138214	high	GSM971957	1.31835	high
TCGA-G4-6627	0.605453	low	GSM972055	1.317647	high
TCGA-A6-2677	1.136158	high	GSM972260	1.316799	high
TCGA-A6-6141	0.727846	low	GSM972375	1.316218	high
TCGA-AA-3939	1.07601	high	GSM972427	1.315141	high
TCGA-A6-2679	1.414963	high	GSM437161	1.314393	high
TCGA-AA-3502	0.739581	low	GSM972303	1.313415	high
TCGA-D5-6533	0.574283	low	GSM972199	1.313373	high
TCGA-AA-3693	1.246251	high	GSM972190	1.312868	high
TCGA-AD-6548	1.246298	high	GSM972421	1.303772	high
TCGA-CM-6170	1.055798	high	GSM437104	1.303111	high
TCGA-AA-3672	1.770488	high	GSM972229	1.30291	high
TCGA-AA-3553	1.396255	high	GSM734143	1.3018	high
TCGA-A6-A56B	2.098913	high	GSM437134	1.299951	high
TCGA-D5-6927	1.273269	high	GSM972516	1.299609	high
TCGA-AA-A00E	0.872685	low	GSM734125	1.296882	high
TCGA-AA-3814	0.86325	low	GSM437271	1.294861	high
TCGA-A6-2676	2.046551	high	GSM437294	1.293663	high
TCGA-AA-A02K	1.214045	high	GSM972385	1.288572	high
TCGA-F4-6570	1.069987	high	GSM972175	1.28808	high
TCGA-DM-A1D9	1.178487	high	GSM972245	1.284889	high
TCGA-AA-3956	0.505838	low	GSM437171	1.282935	high
TCGA-A6-2683	1.340526	high	GSM437246	1.282495	high
TCGA-AA-3713	0.766239	low	GSM972407	1.278892	high
TCGA-AA-3955	0.694486	low	GSM972392	1.275711	high
TCGA-AZ-6598	0.800486	low	GSM972328	1.269281	high
TCGA-CM-6163	0.751876	low	GSM972098	1.266244	high
TCGA-AA-3869	1.378295	high	GSM972118	1.265247	high
TCGA-AA-3538	0.889077	low	GSM437268	1.263521	high
TCGA-DM-A1DA	1.924533	high	GSM972444	1.258726	high
TCGA-AA-3494	0.7729	low	GSM972111	1.257711	high
TCGA-CM-6168	0.734591	low	GSM437220	1.256513	high
TCGA-AA-3851	0.438695	low	GSM734155	1.255783	high
TCGA-A6-6654	1.444616	high	GSM437291	1.252993	high
TCGA-DM-A28E	0.80091	low	GSM972280	1.252897	high
TCGA-AA-3509	0.571072	low	GSM972306	1.251655	high
TCGA-AA-3681	0.732668	low	GSM972182	1.251308	high
TCGA-AA-A00Q	0.825918	low	GSM437185	1.249149	high
TCGA-AM-5820	0.605253	low	GSM972415	1.2489	high
TCGA-CK-6746	1.08769	high	GSM437183	1.247807	high
TCGA-AA-A00W	0.809239	low	GSM972075	1.247681	high
TCGA-AZ-4323	1.525744	high	GSM972259	1.246982	high
TCGA-CM-4746	0.986472	low	GSM437250	1.244891	high
TCGA-AA-3663	0.952995	low	GSM972241	1.244541	high
TCGA-AA-3549	1.06902	high	GSM972267	1.24401	high
TCGA-AA-3561	0.922298	low	GSM437197	1.243634	high
TCGA-G4-6626	1.921399	high	GSM972173	1.242742	high
TCGA-A6-2681	0.938573	low	GSM972274	1.242394	high
TCGA-AY-6197	0.580036	low	GSM972078	1.239868	high
TCGA-AY-6386	0.666047	low	GSM972203	1.235278	high
TCGA-AU-3779	1.399219	high	GSM437305	1.23444	high

TCGA-AA-A00F	0.842375	low
TCGA-AA-3866	1.100994	high
TCGA-AA-A01K	0.961474	low
TCGA-AA-3544	1.220659	high
TCGA-A6-A567	1.470038	high
TCGA-CM-6676	0.743843	low
TCGA-CM-4752	0.964482	low
TCGA-NH-A8F7	0.624376	low
TCGA-AA-A02W	1.501612	high
TCGA-AA-3524	0.83337	low
TCGA-AY-A69D	0.646911	low
TCGA-AA-3673	0.919191	low
TCGA-AA-3855	0.662206	low
TCGA-AA-3952	0.650317	low
TCGA-AA-3532	1.210335	high
TCGA-AA-3989	0.83524	low
TCGA-AA-3530	0.597551	low
TCGA-NH-A5IV	1.688916	high
TCGA-CK-4947	0.829247	low
TCGA-AA-3554	1.014522	high
TCGA-D5-5538	0.966689	low
TCGA-AA-3526	1.128061	high
TCGA-CA-5255	0.587998	low
TCGA-AA-3977	1.325921	high
TCGA-AA-A01F	0.941351	low
TCGA-D5-5540	2.63627	high
TCGA-5M-AAT4	1.76982	high
TCGA-AA-3864	1.271876	high
TCGA-CM-6161	0.87019	low
TCGA-AA-3819	0.979256	low
TCGA-AA-3861	0.648704	low
TCGA-D5-6926	1.501961	high
TCGA-AA-3531	1.109016	high
TCGA-AA-3685	1.214741	high
TCGA-AA-3712	0.956798	low
TCGA-AA-3941	0.701712	low
TCGA-A6-5660	0.924465	low
TCGA-A6-6652	1.054402	high
TCGA-CM-5868	1.116767	high
TCGA-CM-6164	1.391663	high
TCGA-AZ-6606	0.827484	low
TCGA-DM-A1HA	1.776707	high
TCGA-G4-6323	0.482136	low
TCGA-AA-3542	1.070658	high
TCGA-D5-6924	1.677213	high
TCGA-QG-A5YV	0.978959	low
TCGA-AA-3688	1.151405	high
TCGA-AA-3980	1.160627	high
TCGA-AA-3525	0.494147	low
TCGA-AY-4070	1.189413	high
TCGA-AA-3495	0.652969	low
TCGA-D5-6531	2.000495	high
TCGA-CA-5254	1.598292	high
TCGA-QL-A97D	1.194275	high
TCGA-AA-A02R	3.825856	high
TCGA-F4-6805	1.047797	high
TCGA-AA-3696	0.625164	low
TCGA-A6-5662	0.79523	low

GSM437219	1.232306	high
GSM437202	1.228563	high
GSM972374	1.228454	high
GSM972254	1.227293	high
GSM437143	1.227186	high
GSM734156	1.223851	high
GSM972255	1.221369	high
GSM972325	1.218319	high
GSM437182	1.217598	high
GSM437322	1.214662	high
GSM972006	1.213719	high
GSM972264	1.212608	high
GSM972231	1.212596	high
GSM437200	1.211912	high
GSM972438	1.206561	high
GSM972393	1.205826	high
GSM972521	1.204492	high
GSM437266	1.203022	high
GSM437159	1.197562	high
GSM734119	1.193979	high
GSM437137	1.193447	high
GSM437310	1.192811	high
GSM972293	1.187158	high
GSM437205	1.18502	high
GSM437146	1.183826	high
GSM972215	1.180544	high
GSM972214	1.180439	high
GSM437119	1.180128	high
GSM972043	1.174149	high
GSM437297	1.171828	high
GSM437103	1.171404	high
GSM972218	1.170536	high
GSM437258	1.170384	high
GSM972388	1.165803	high
GSM972391	1.162488	high
GSM972146	1.161243	high
GSM734115	1.160894	high
GSM437093	1.160218	high
GSM972017	1.157636	high
GSM971982	1.154522	high
GSM437098	1.153922	high
GSM972183	1.15088	high
GSM972289	1.150527	high
GSM437264	1.148705	high
GSM437094	1.147573	high
GSM437279	1.147034	high
GSM437317	1.146071	high
GSM972371	1.143896	high
GSM972031	1.143334	high
GSM437165	1.141752	high
GSM734168	1.140026	high
GSM437184	1.13871	high
GSM437248	1.137761	high
GSM734147	1.137675	high
GSM437302	1.133713	high
GSM437199	1.133578	high
GSM972003	1.127019	high
GSM972234	1.126845	high

TCGA-AZ-6601	1.668623	high
TCGA-DM-A1D4	0.460845	low
TCGA-RU-A8FL	0.680094	low
TCGA-AA-3975	1.029322	high
TCGA-F4-6460	1.093738	high
TCGA-AZ-4308	1.210857	high
TCGA-AA-3972	0.811414	low
TCGA-AA-3858	1.446891	high
TCGA-AA-3679	1.088801	high
TCGA-AY-5543	0.921828	low
TCGA-AU-6004	0.985486	low
TCGA-A6-6137	0.43924	low
TCGA-AA-3860	0.803027	low
TCGA-AA-3986	0.997369	low
TCGA-CM-6165	0.414835	low
TCGA-AA-3520	1.015219	high
TCGA-G4-6625	0.901459	low
TCGA-DM-A28M	0.514478	low
TCGA-D5-6920	0.530975	low
TCGA-AA-A02O	1.195585	high
TCGA-AA-A02E	1.475171	high
TCGA-AA-3710	1.130608	high
TCGA-AZ-4614	1.239826	high
TCGA-G4-6320	1.116698	high
TCGA-AA-3856	0.818291	low
TCGA-A6-6138	0.962642	low
TCGA-A6-2685	1.105311	high
TCGA-QG-A5YX	0.356556	low
TCGA-AY-A54L	0.962626	low
TCGA-A6-2671	2.009183	high
TCGA-NH-A8F8	1.333526	high
TCGA-D5-6932	1.804453	high
TCGA-AA-3675	0.664589	low

GSM972398	1.126253	high
GSM972279	1.126156	high
GSM972023	1.126012	high
GSM437101	1.125882	high
GSM972158	1.125409	high
GSM437262	1.125235	high
GSM437167	1.124238	high
GSM972073	1.124013	high
GSM972167	1.122244	high
GSM972244	1.120768	high
GSM972494	1.119531	high
GSM971978	1.116118	high
GSM972418	1.115952	high
GSM972087	1.115815	high
GSM972377	1.114994	high
GSM972119	1.114835	high
GSM972497	1.110891	high
GSM437243	1.110383	high
GSM437111	1.110033	high
GSM437273	1.109719	high
GSM734116	1.10899	high
GSM972155	1.106811	high
GSM437308	1.105383	high
GSM437096	1.105352	high
GSM972237	1.102925	high
GSM972449	1.101631	high
GSM972160	1.099374	high
GSM972128	1.097176	high
GSM972382	1.088633	high
GSM971959	1.088259	high
GSM972411	1.087049	high
GSM972390	1.086388	high
GSM972144	1.085533	high
GSM972083	1.085526	high
GSM972475	1.084454	high
GSM972077	1.083563	high
GSM437263	1.081274	high
GSM437295	1.081269	high
GSM734165	1.080226	high
GSM972468	1.077052	high
GSM972282	1.07539	high
GSM734139	1.075097	high
GSM437235	1.074945	high
GSM437156	1.074904	high
GSM972014	1.074745	high
GSM972154	1.074621	high
GSM972348	1.0743	high
GSM972490	1.07359	high
GSM972141	1.072489	high
GSM972169	1.067771	high
GSM972142	1.066096	high
GSM972013	1.065944	high
GSM972257	1.065636	high
GSM972238	1.063506	high
GSM972498	1.062324	high
GSM437277	1.062314	high
GSM972465	1.061645	high
GSM437132	1.060283	high

GSM734154	1.056914	high
GSM971985	1.056381	high
GSM972269	1.056341	high
GSM437121	1.056111	high
GSM972029	1.05343	high
GSM972358	1.053286	high
GSM972368	1.052983	high
GSM437298	1.051569	high
GSM734141	1.047949	high
GSM437139	1.047236	high
GSM972263	1.046741	high
GSM972440	1.044179	high
GSM972132	1.044007	high
GSM972248	1.043865	high
GSM972222	1.041463	high
GSM734113	1.041219	high
GSM972195	1.040796	high
GSM972036	1.040399	high
GSM437194	1.038411	high
GSM972344	1.03397	high
GSM972139	1.03363	high
GSM437252	1.033483	high
GSM971977	1.033407	high
GSM972336	1.033106	high
GSM972027	1.030384	high
GSM734124	1.028555	high
GSM972424	1.027529	high
GSM437109	1.024431	high
GSM437105	1.023198	high
GSM972106	1.022	high
GSM972483	1.021801	high
GSM437177	1.021474	high
GSM972310	1.020147	high
GSM972431	1.019962	high
GSM972148	1.019532	high
GSM972035	1.018427	high
GSM437122	1.017881	high
GSM972110	1.017277	high
GSM972243	1.016509	high
GSM972364	1.015538	high
GSM437113	1.013463	high
GSM972300	1.013089	high
GSM972370	1.012868	high
GSM437110	1.011948	high
GSM972268	1.01194	high
GSM972053	1.01089	high
GSM972351	1.010789	high
GSM437234	1.009804	high
GSM972107	1.007543	high
GSM972472	1.004551	high
GSM734140	1.004452	high
GSM437259	1.002228	high
GSM972037	1.000638	high
GSM437296	0.998869	high
GSM972505	0.998796	high
GSM972482	0.998037	high
GSM437244	0.999592	high
GSM972436	0.997274	low

GSM972386	0.996961	low
GSM972517	0.994905	low
GSM734135	0.994833	low
GSM972256	0.993964	low
GSM437178	0.993384	low
GSM972414	0.987715	low
GSM972297	0.987103	low
GSM972196	0.985819	low
GSM972161	0.985214	low
GSM437214	0.985155	low
GSM971992	0.985077	low
GSM972307	0.984888	low
GSM972200	0.984824	low
GSM972044	0.983675	low
GSM972311	0.982695	low
GSM972022	0.982318	low
GSM972347	0.981968	low
GSM437115	0.981129	low
GSM437180	0.979355	low
GSM734153	0.979003	low
GSM972378	0.978178	low
GSM972442	0.97541	low
GSM971979	0.974844	low
GSM972296	0.974081	low
GSM971964	0.973077	low
GSM437100	0.97166	low
GSM972402	0.971364	low
GSM972140	0.966682	low
GSM972056	0.966276	low
GSM437285	0.965716	low
GSM972019	0.965066	low
GSM437172	0.964628	low
GSM972033	0.964495	low
GSM972342	0.963729	low
GSM972065	0.961234	low
GSM734163	0.959793	low
GSM972249	0.957932	low
GSM437166	0.95771	low
GSM437140	0.957392	low
GSM437136	0.952588	low
GSM437215	0.951106	low
GSM972063	0.950344	low
GSM437281	0.948281	low
GSM972373	0.947449	low
GSM972038	0.945995	low
GSM971994	0.944839	low
GSM972441	0.942918	low
GSM437191	0.942628	low
GSM972071	0.941289	low
GSM437125	0.940733	low
GSM437230	0.939157	low
GSM972187	0.937467	low
GSM971984	0.936576	low
GSM972153	0.936502	low
GSM437099	0.935419	low
GSM972435	0.93531	low
GSM972486	0.934617	low
GSM972323	0.934271	low

GSM437168	0.934135	low
GSM972021	0.933383	low
GSM972506	0.932749	low
GSM972048	0.932369	low
GSM437228	0.932274	low
GSM972093	0.929934	low
GSM972469	0.929559	low
GSM972518	0.928394	low
GSM972450	0.923987	low
GSM437208	0.923674	low
GSM972258	0.923539	low
GSM437141	0.923524	low
GSM972129	0.923098	low
GSM972240	0.922416	low
GSM437155	0.921689	low
GSM972299	0.920987	low
GSM437108	0.920575	low
GSM437192	0.917729	low
GSM437222	0.917659	low
GSM734164	0.917259	low
GSM972191	0.917257	low
GSM972403	0.915692	low
GSM437312	0.913379	low
GSM972227	0.913035	low
GSM734137	0.91223	low
GSM972474	0.908797	low
GSM972252	0.908794	low
GSM437158	0.90646	low
GSM437144	0.905408	low
GSM437306	0.903209	low
GSM972346	0.902319	low
GSM972362	0.902283	low
GSM972109	0.901666	low
GSM971983	0.901423	low
GSM972266	0.896776	low
GSM972092	0.895563	low
GSM972455	0.894364	low
GSM437225	0.891065	low
GSM972332	0.890958	low
GSM437206	0.889342	low
GSM437227	0.887436	low
GSM437157	0.8871	low
GSM437247	0.886477	low
GSM972127	0.884737	low
GSM437211	0.882638	low
GSM972272	0.882198	low
GSM734126	0.881755	low
GSM972198	0.881494	low
GSM972080	0.877267	low
GSM972387	0.876393	low
GSM972025	0.873225	low
GSM972437	0.872912	low
GSM734121	0.872581	low
GSM972285	0.87182	low
GSM437299	0.871098	low
GSM972302	0.870626	low
GSM972273	0.868025	low
GSM437224	0.865187	low

GSM734146	0.862423	low
GSM972230	0.861571	low
GSM437151	0.861237	low
GSM972493	0.861134	low
GSM437107	0.860631	low
GSM972096	0.859225	low
GSM972130	0.859077	low
GSM972366	0.858288	low
GSM437282	0.856031	low
GSM971974	0.855936	low
GSM437221	0.853414	low
GSM972510	0.851577	low
GSM437261	0.851574	low
GSM734172	0.851499	low
GSM437181	0.85064	low
GSM972514	0.847108	low
GSM972454	0.847078	low
GSM734161	0.844497	low
GSM734152	0.844092	low
GSM972278	0.844	low
GSM972394	0.843416	low
GSM437275	0.843193	low
GSM437164	0.84303	low
GSM972295	0.842713	low
GSM972312	0.842582	low
GSM437260	0.841426	low
GSM972062	0.839969	low
GSM734169	0.83996	low
GSM972477	0.839468	low
GSM437303	0.838897	low
GSM734117	0.838667	low
GSM971988	0.838091	low
GSM972058	0.836526	low
GSM734112	0.835559	low
GSM972192	0.833053	low
GSM972331	0.832348	low
GSM972511	0.830631	low
GSM971969	0.829537	low
GSM437237	0.829481	low
GSM437162	0.829384	low
GSM972513	0.829168	low
GSM437289	0.827475	low
GSM972242	0.826722	low
GSM437150	0.824544	low
GSM972204	0.818136	low
GSM972137	0.81751	low
GSM972512	0.816352	low
GSM972001	0.816071	low
GSM437129	0.815665	low
GSM972337	0.815314	low
GSM972133	0.810547	low
GSM972412	0.808723	low
GSM437127	0.808668	low
GSM972049	0.807465	low
GSM734129	0.806796	low
GSM972177	0.805315	low
GSM972399	0.802299	low
GSM972277	0.799459	low

GSM972028	0.798407	low
GSM972352	0.797864	low
GSM972168	0.796268	low
GSM972197	0.795723	low
GSM972178	0.795511	low
GSM972372	0.795391	low
GSM437169	0.794672	low
GSM972379	0.794336	low
GSM972432	0.793368	low
GSM437255	0.79264	low
GSM971990	0.792163	low
GSM972125	0.791077	low
GSM972459	0.788966	low
GSM734171	0.788652	low
GSM972041	0.787542	low
GSM971993	0.783919	low
GSM972032	0.782422	low
GSM972034	0.782138	low
GSM437210	0.781309	low
GSM734150	0.780821	low
GSM734157	0.78036	low
GSM437321	0.779599	low
GSM971975	0.779394	low
GSM972333	0.778726	low
GSM734120	0.778173	low
GSM437217	0.777334	low
GSM972228	0.777274	low
GSM437218	0.776446	low
GSM972478	0.776373	low
GSM437323	0.774799	low
GSM972114	0.77394	low
GSM972149	0.773771	low
GSM437114	0.773618	low
GSM972226	0.773024	low
GSM437324	0.77163	low
GSM437123	0.769575	low
GSM972460	0.767026	low
GSM437269	0.766256	low
GSM972052	0.764361	low
GSM972520	0.764259	low
GSM972211	0.761464	low
GSM972495	0.756456	low
GSM437147	0.756293	low
GSM972000	0.752656	low
GSM972086	0.752033	low
GSM437309	0.75172	low
GSM437195	0.749286	low
GSM972201	0.749264	low
GSM972321	0.747035	low
GSM437233	0.746859	low
GSM437120	0.746698	low
GSM972005	0.746063	low
GSM437149	0.745368	low
GSM972429	0.74504	low
GSM734158	0.744526	low
GSM734173	0.739806	low
GSM437292	0.739114	low
GSM972138	0.736684	low

GSM734142	0.735697	low
GSM734138	0.732218	low
GSM972219	0.732113	low
GSM972164	0.731611	low
GSM972136	0.73088	low
GSM972456	0.727547	low
GSM972330	0.725899	low
GSM972085	0.725814	low
GSM437095	0.725058	low
GSM972315	0.721848	low
GSM972384	0.721393	low
GSM734174	0.720755	low
GSM437232	0.718039	low
GSM437253	0.717661	low
GSM972509	0.717169	low
GSM972316	0.716258	low
GSM972185	0.716238	low
GSM971999	0.715061	low
GSM437187	0.709034	low
GSM734167	0.70798	low
GSM972099	0.707655	low
GSM972405	0.707034	low
GSM972461	0.706078	low
GSM734149	0.700268	low
GSM437131	0.698985	low
GSM972159	0.698333	low
GSM972250	0.697997	low
GSM972270	0.697204	low
GSM972135	0.696497	low
GSM437257	0.695104	low
GSM972100	0.693515	low
GSM972515	0.692623	low
GSM972060	0.691682	low
GSM437288	0.690141	low
GSM972134	0.688006	low
GSM972355	0.68368	low
GSM972016	0.682114	low
GSM971972	0.680453	low
GSM972166	0.680148	low
GSM972030	0.679784	low
GSM972443	0.679673	low
GSM972081	0.679469	low
GSM972223	0.677843	low
GSM972091	0.677548	low
GSM437128	0.676023	low
GSM972396	0.675496	low
GSM972338	0.674615	low
GSM972383	0.673637	low
GSM972074	0.672804	low
GSM972287	0.670437	low
GSM972381	0.670329	low
GSM437320	0.670039	low
GSM437311	0.668054	low
GSM734136	0.667487	low
GSM972423	0.665628	low
GSM734127	0.662726	low
GSM972012	0.662637	low
GSM972339	0.660139	low

GSM972094	0.659016	low
GSM437238	0.658219	low
GSM972453	0.657054	low
GSM972496	0.656363	low
GSM972298	0.656239	low
GSM972007	0.655279	low
GSM972239	0.6541	low
GSM972304	0.651841	low
GSM972233	0.649719	low
GSM437176	0.647249	low
GSM972320	0.645563	low
GSM972188	0.644421	low
GSM971989	0.644246	low
GSM972221	0.643857	low
GSM972174	0.643592	low
GSM971987	0.640178	low
GSM972488	0.64007	low
GSM971971	0.638846	low
GSM437154	0.637314	low
GSM972317	0.628261	low
GSM972473	0.628058	low
GSM437170	0.625977	low
GSM437274	0.624913	low
GSM437251	0.624895	low
GSM972508	0.622341	low
GSM972172	0.614214	low
GSM734144	0.60683	low
GSM972480	0.605954	low
GSM972147	0.604315	low
GSM972150	0.603552	low
GSM734175	0.602279	low
GSM734132	0.60082	low
GSM972416	0.600216	low
GSM437138	0.5994	low
GSM971965	0.599266	low
GSM734159	0.598929	low
GSM972101	0.597217	low
GSM972447	0.595132	low
GSM972151	0.59442	low
GSM972102	0.592179	low
GSM972045	0.592134	low
GSM972115	0.591618	low
GSM972359	0.587893	low
GSM972050	0.57903	low
GSM972202	0.575235	low
GSM972507	0.573946	low
GSM972152	0.57204	low
GSM437193	0.570448	low
GSM972261	0.568508	low
GSM972503	0.566549	low
GSM972340	0.566096	low
GSM972457	0.565593	low
GSM972090	0.560545	low
GSM972292	0.56003	low
GSM972046	0.558936	low
GSM972326	0.553331	low
GSM972040	0.55324	low
GSM972489	0.550873	low

GSM972165	0.548738	low
GSM437097	0.547644	low
GSM972156	0.547025	low
GSM972020	0.540617	low
GSM972319	0.537815	low
GSM972417	0.536583	low
GSM972291	0.530141	low
GSM972404	0.526858	low
GSM972434	0.526216	low
GSM972206	0.509169	low
GSM972522	0.508417	low
GSM971976	0.504254	low
GSM972463	0.503616	low
GSM972145	0.50321	low
GSM972329	0.498974	low
GSM734166	0.497644	low
GSM972301	0.486719	low
GSM972350	0.483651	low
GSM971967	0.480918	low
GSM972309	0.47743	low
GSM972290	0.476808	low
GSM972246	0.476126	low
GSM972253	0.474631	low
GSM437231	0.468243	low
GSM971980	0.466068	low
GSM971960	0.465052	low
GSM972124	0.463604	low
GSM437316	0.460121	low
GSM972117	0.455492	low
GSM972439	0.447149	low
GSM972487	0.446973	low
GSM971995	0.446836	low
GSM972305	0.444869	low
GSM972042	0.425829	low
GSM734134	0.423529	low
GSM972084	0.417687	low
GSM972327	0.417372	low
GSM972088	0.401006	low
GSM971998	0.386282	low
GSM972009	0.382713	low
GSM972116	0.380571	low
GSM972400	0.361668	low
GSM972163	0.355947	low
GSM437283	0.353908	low
GSM1681356	0.333756	low
GSM437304	0.33207	low
GSM972189	0.331449	low
GSM437216	0.318908	low
GSM972070	0.299893	low
GSM1681359	0.285672	low
GSM1681360	0.27401	low
GSM1681371	0.269427	low
GSM1681369	0.229679	low
GSM972357	0.220847	low
GSM1681363	0.219565	low
GSM1681354	0.216379	low
GSM1681370	0.215541	low
GSM1681365	0.212637	low

GSM1681367	0.203349	low
GSM1681355	0.198606	low
GSM1681362	0.196104	low
GSM1681368	0.188589	low
GSM1681353	0.186485	low
GSM1681361	0.18205	low
GSM1681357	0.177764	low
GSM1681364	0.164465	low
