# Supplementary material for: Construction and validation of an immune-related genes prognostic index (IRGPI) model in colon cancer
Source: Front Endocrinol (Lausanne). 2022 Nov 9;13:963382. doi: 10.3389/fendo.2022.963382 (PMC9682206; doi:10.3389/fendo.2022.963382)
Supplement: Supplementary Material S4 — Univariate and multivariate cox regression analysis of the correlation between age, T stage, N stage, M stage, clinical stage andIRGPI and survival. [file DataSheet_4.pdf]

Table

Supplementary material S4. Univariate analysis and multivariate cox regression analysis of the correlation between clinical characteristics, IRGPI and survival.

a.Univariate anlaysis

Characteristic	HR	95% CI	p value
Age	1.03	1.01-1.06	0.006
Gender	1.00	0.61-1.62	0.996
T	3.23	1.97-5.29	0.000
N	2.14	1.61-2.85	0.000
M	5.08	3.06-8.42	0.000
Stage	2.46	1.85-3.26	0.000
IRGPI	1.96	1.40-2.76	0.000

b.Multivariate cox regression analysis

Characteristic	HR	HR.95L	p value
Age	1.04	1.02-1.06	0.001
T	1.75	0.98-3.15	0.060
N	1.12	0.68-1.85	0.649
M	1.45	0.47-4.44	0.514
Stage	1.78	0.78-4.03	0.169
IRGPI	1.79	1.20-2.65	0.004
